# Provably secure and lightweight blockchain based cross hospital authentication scheme for IoMT-based healthcare

**DOI:** 10.1038/s41598-025-90219-5

**Published:** 2025-02-22

**Authors:** Qi Xie, Zixuan Ding

**Affiliations:** 1 Key Laboratory of Cryptography of Zhejiang Province, Hangzhou Normal University, Hangzhou, 311121 China; 2 College of Cryptology and Cyber Science, Nankai University, Tianjin, 300350 China

**Keywords:** Authentication protocol, Internet of Medical Things, Blockchain, Healthcare, Cross-hospital, Decentralization, Health care, Health services

## Abstract

Portable devices and sensors-based Internet of Medical Things (IoMT) healthcare can remotely detect patients’ physiological data and provide first-class healthcare services. However, the high privacy and sensitivity of medical data make IoMT healthcare systems vulnerable to various attacks. While numerous authentication protocols have been introduced in recent years to guarantee authorized access, these schemes continue to face challenges such as privacy disclosure, untraceability of malicious behavior, insufficient cross-hospital access, and concerns related to single points of failure and trust. To address these issues, we propose a Double Anonymity Strategy to hide identities between doctors and the patients while allowing the authorized party to track their malicious behavior, enhance users’ privacy and track malicious users. Our approach leverages the advantages of blockchain, such as decentralization, and replaces trusted third parties with smart contracts for efficient and automatic identity authentication. Additionally, we introduce a cross-hospital authentication scheme that incorporates three-factor secrecy, ensuring that even if any two of the three factors (device, biometric information and password) are compromised, the security of the proposed scheme will not be affected. The security of our scheme is formally proven under the random oracle model, which formally measures that the probability of an adversary breaking the scheme is negligible. We also provide informal security analysis showing that our scheme prevents privacy breaches, achieves decentralization, and addresses existing various attacks. Furthermore, through simulation of the proposed scheme and comparison with related works, we demonstrate that our scheme achieves 23% to 87% reduction in computational cost while maintaining higher security properties.

## Introduction

With the aging of the global population and the continuous increase of chronic patients, changing traditional healthcare methods has become a consensus. Thanks to the rapid development and application of emerging technologies, such as wearable devices, Internet of Things, and mobile Internet, IoMT-based healthcare dynamically connects devices and medical-related participants, intelligently builds a convenient, efficient, and personalized medical system. Compared with the traditional medical model, IoMT-based healthcare system provides more professional medical services for patients and improves doctors’ diagnoses, which represents the future development direction. However, although mobile communication is convenient, it also suffers from several security risks, which not only reveal doctors’ and patients’ privacy but also pose a threat to patients’ health and system security. To resist them, research works have been continuously conducted to improve the authentication protocols for IoMT-based healthcare.

Many password-based authentication schemes for medical scenarios continue to emerge, which do not require additional hardware devices. However, unreasonable verification strategies or password storage methods may lead to serious password guessing attacks and pre-computation attacks^1^. To enhance security, biometric key was introduced as an authentication factor^2^. In addition, several cryptographic primitives, such as Chebyshev chaotic map (The Chebyshev chaotic map is a mathematical function derived from Chebyshev polynomials, which exhibit chaotic behavior under certain conditions), RSA (Rivest-Shamir-Adleman is an asymmetric cryptographic algorithm used for secure data transmission. It relies on the mathematical difficulty of factoring large composite numbers), Elliptic Curve Cryptography (ECC, which is an asymmetric cryptographic technique based on the algebraic structure of elliptic curves over finite fields. ECC provides the same level of security as traditional systems like RSA but with much smaller key sizes, making it more efficient in terms of computational power and memory usage), and bilinear pairing (Bilinear pairing is a mathematical operation on two groups that maps them into a third group, preserving certain linear properties) are used in authentication protocols to ensure perfect forward secrecy and session key secrecy. Among them, bilinear pairing is seldomly adopted due to high computational cost. Besides, the above authentication schemes must rely on a Trusted Third Party (TTP) to complete authentication and key agreement, while the communication is limited to a cluster centered on the TTP. Furthermore, privacy protection and tracking malicious behavior seem to mutually exclusive to each other and hard to be addressed.

Blockchain serves as a decentralized and tamper-proof distributed shared ledger and database with traceability. With the above properties, blockchain balances the information asymmetry in some application scenarios, and realizes the trust and concerted action of multiple participants. As a technology closely combined with blockchain, smart contracts are computer programs that support automagical execution. The rules of smart contract execution are transparent and cannot be maliciously tampered. Therefore, it can replace TTP as an honest transponder. Recently, blockchain and smart contracts have been widely valued and applied to medical authentication protocols to solve the above problems.

### Related works

In 2016, Mettler^3^ proposed the idea of using blockchain instead of TTP in medical field to eliminate the limitation of centralized storage. However, he did not design a specific and realizable scheme. Next year, Sullivan and Burger^4^ demonstrated the idea of widely applying blockchain to citizenship authentication from the aspects of law, policy, and technology. Srivastava et al.^5^ designed a data sharing scheme based on blockchain for patient IoT devices. Wearable devices are used to monitor patients, while the events are recorded by smart contracts on the public blockchain to support traceability. However, the proposed idea lacks of specific protocol and cannot meet the instantiation requirements. Dwivedi et al.^6^ proposed a decentralized privacy-preserving blockchain for health IoT, which uses ring signature to ensure users’ anonymity and authenticity, but excessive expenses remain the major obstacle to practicality. In 2020, Yazdinejad et al.^7^ designed an efficient authentication method based on blockchain for distributed hospital networks. But we found that the scheme lacks of necessary security in the sense that it cannot prevent impersonation attacks. In addition, the scheme based on public blockchain cannot guarantee privacy protection and anonymity in the authentication process. Xiang et al.^8^ designed a user authentication protocol based on blockchain for E-healthcare. They treat the blockchain as a trusted distributed ledger without considering its application in scaling the communication cluster. Deebak et al.^9^ proposed an authentication protocol for cloud-assisted healthcare systems. Jia et al.^10^ presented an overarching framework for a blockchain-integrated IoMT system within the fog computing architecture, encompassing diverse entities. The system incorporates an ECC-based authentication scheme focused on preserving user privacy in communications between end users and fog nodes. Saha et al.^11^ proposed an authentication and access control for healthcare based on blockchain. Private blockchain stores patients’ encrypted information. However, the key is shared by trusted hospital nodes, implying that it cannot protect the patients’ privacy and cannot resist known key attack. Addressing the challenge of cross-domain authentication within the PKI system, Chen et al.^12^ introduced an innovative solution that separates the storage and control layers. They employ a multi-layered Merkle hash tree structure to handle extensive identity data efficiently and devise a streamlined correctness validation protocol to enhance response times. The incorporation of a zero-knowledge proof algorithm, coupled with constraints on data types within the distributed ledger, ensures the protection of users’ privacy during cross-domain authentication. Javed et al.^13^ proposed a blockchain based identity management system, which supports service providers and patients to authenticate and identify transparently and safely. Blockchain is used to build indexes and provide distributed identity management services. However, the authentication process still needs the participation of a TTP and the registry. Nguyen et al.^14^ designed a blockchain and Mobile-Edge Computing based framework for IoMT network, which realizes data sharing and data offloading in the distributed hospital network. Furthermore, the authentication strategy based on smart contracts is combined with Mobile Edge Computing (MEC) to realize access control of users without central authorization. Egala et al.^15^ designed a blockchain-based framework for IoMT, which realizes decentralized Electrical Health Records (EHR) and automated services based on smart contracts. To raise security, device authentication and patient anonymity rely on distributed selective ring-based access control strategy.

In 2023, Tomar et al.^16^ proposed a protocol named Blockchain-based IoMT Authenticated Key Exchange (BIoMTAKE) key exchange to create a distributed environment using Hyperledger Fabric for private/consortium blockchains. This protocol eliminates the necessity for a singular trusted authority and ensures secure data access from IoMT devices. Prior to data sharing or access within the distributed healthcare system, the BIoMTAKE scheme establishes a secured shared session for authenticated devices to prevent unauthorized entry. Wazid and Gope^17^ introduced a novel blockchain-powered access control and key management protocol, named "BACKM-EHA," for IoMT-based e-healthcare systems. Through rigorous security analyses conducted in accordance with the Real-Or-Random model, the robustness of BACKM-EHA against various potential attacks is demonstrated. Kumar et al. introduce the RAPCHI protocol^18^, which has important features such as establishing authentication and key agreement between patients, cloud servers, and doctors, forming session keys without storing data in the cloud database, resisting various security threats, and meeting multiple security requirements. Chen et al.^19^ propose a new provably secure remote patient monitoring healthcare system protocol to address data security issues in IoMT systems. The protocol incorporates user biometric information and is proven secure using the random oracle model. Compared to other similar works, it offers more protection and has advantages in execution and communication costs.

In 2024, Ali et al.^20^ mainly discuss the integration of 5G edge computing in IoMT. They highlight that while it brings the prospect of decentralized healthcare services, it also raises significant security issues, particularly concerning data integrity and access control. The paper proposes an innovative encryption solution called Online/Offline Remote Signcryption (O2RSC), which effectively addresses these limitations by providing integrity and access control services while reducing computational operations in the online mode. Zhang et al.^21^ point out that recent certificateless signcryption (CLSC) schemes in IoMT are vulnerable to attacks. Based on elliptic curve cryptography and specific security assumptions, including entities in the system model such as sensor devices, key generation centers, cloud servers, and doctors, as well as the corresponding security model and security objectives, they propose a pairing-free CLSC scheme for secure data transmission. This scheme features public verifiability, low computational and communication overhead, and can resist both Type I and Type II attacks. Mahmood et al.^22^ explore a cost-effective authentication solution (CAS) for 6G-enabled Artificial Intelligence of Medical Things (AIoMT) healthcare applications. In 6G-enabled AIoMT healthcare applications, the security and seamlessness of information exchange are major challenges. The authors design the CAS protocol using simple cryptographic primitives to reduce development complexity, capable of defending against network and physical threats. Performance comparisons show that it has advantages in enhancing security and cost-effectiveness. Singh and Dash^23^ point out that the Internet of Medical Things (IoMT) faces numerous challenges related to data processing, storage, management, and security. For example, third-party authentication can present single points of failure and data loss risks, while centralized architectures are vulnerable to attacks. Although smart contract authentication eliminates the need for third parties, it is susceptible to Sybil attacks and 51% attacks. Additionally, issues related to physical layer security and scalability remain unresolved. The authors employ PUF, fuzzy extractors, and smart contract-enabled Inter Planetary File System (IPFS) clusters to achieve two-factor authentication for users and devices, providing security at both the physical and network layers. This ensures data confidentiality, integrity, and anonymity. They conduct both formal and informal security analyses and performance evaluations. Table 1 provides a summary of the most relevant technologies for IoMT authentication protocols and their advantages and disadvantages. Xie et al.^24^ note that IoMT data is vulnerable to cyberattacks as existing multi-server authentication protocols lack continuous monitoring. They propose multi-server authentication scheme with integrated monitoring, which combines three-factor-based static authentication (TFSA) and deep learning-based continuous authentication (DLCA). TFSA ensures privacy and security, and DLCA validates users via behavior analysis, with both components demonstrating strong security and feasibility. Qu et al.^25^ note that Electrocardiogram (ECG) leakage is common in the context of arrhythmia detection, and classical blockchain fails to safeguard ECG data in the quantum era. They propose QADS (Quantum Arrhythmia Detection System), a smart healthcare system for arrhythmia. It uses a quantum blockchain for secure data handling and a quantum neural network. Rehman et al.^26^ note that the digital transformation of the healthcare industry makes patient data accessible, and blockchain applications in healthcare have drawbacks. They propose an IoMT-based hybrid blockchain architecture. This architecture combines Ethereum and Hyperledger Fabric blockchains with SQLite, introduces access control, and uses machine learning algorithms, and its performance is validated by the M/M/1 queuing model.

**Table 1:** Summary of related schemes.

Schemes	Years	Techniques	Advantages	Limitations (Not Achieve)
^7^	2020	${T}_{8}$(No implementation details provided)	${A}_{4}$, ${A}_{7}$, ${A}_{8}$, ${A}_{9}$, ${RA}_{2}$, ${RA}_{4}$, ${RA}_{5}$, ${RA}_{6}$, ${RA}_{7}$,${RA}_{10}$	${A}_{1}$, ${A}_{2}$, ${A}_{3}$, ${A}_{5}$, ${A}_{6}$, ${RA}_{1}$, ${RA}_{3}$, ${RA}_{5}$, ${RA}_{8}$,${RA}_{9}$
^8^	2020	${T}_{2}$, ${T}_{4}$,${T}_{6}$	${A}_{4}$, ${RA}_{2}$, ${RA}_{6}$, ${RA}_{7}$, ${RA}_{9}$,${RA}_{10}$	${A}_{1}$, ${A}_{2}$, ${A}_{3}$, ${A}_{5}$, ${A}_{6}$, ${A}_{7}$, ${A}_{8}$, ${A}_{9}$, ${RA}_{1}$, ${RA}_{2}$, ${RA}_{3}$, ${RA}_{4}$, ${RA}_{5}$, ${RA}_{6}$,${RA}_{8}$
^10^	2022	${T}_{4}$, ${T}_{5}$, ${T}_{6}$,${T}_{7}$	${A}_{1}$, ${A}_{2}$, ${A}_{4}$, ${A}_{5}$, ${A}_{9}$, ${RA}_{1}$, ${RA}_{2}$, ${RA}_{3}$, ${RA}_{5}$, ${RA}_{6}$, ${RA}_{7}$, ${RA}_{8}$, ${RA}_{9}$,${RA}_{10}$	${A}_{3}$, ${A}_{6}$, ${A}_{7}$,${A}_{8}$
^12^	2020	${T}_{4}$, ${T}_{6}$,${T}_{7}$	${A}_{2}$, ${A}_{3}$, ${A}_{4}$, ${A}_{5}$, ${RA}_{2}$, ${RA}_{3}$, ${RA}_{5}$, ${RA}_{7}$, ${RA}_{8}$, ${RA}_{9}$,${RA}_{10}$	${A}_{1}$, ${A}_{6}$, ${A}_{7}$, ${A}_{8}$, ${A}_{9}$, ${RA}_{1}$,${RA}_{6}$
^14^	2021	${T}_{3}$, ${T}_{6}$,${T}_{7}$	${A}_{7}$, ${A}_{8}$, ${A}_{9}$, ${RA}_{2}$, ${RA}_{3}$, ${RA}_{5}$, ${RA}_{7}$,${RA}_{9}$	${A}_{1}$, ${A}_{2}$, ${A}_{3}$, ${A}_{4}$, ${A}_{5}$, ${A}_{6}$, ${RA}_{1}$, ${RA}_{2}$, ${RA}_{4}$, ${RA}_{6}$, ${RA}_{8}$,${RA}_{10}$
^18^	2022	${T}_{3}$, ${T}_{6}$	${A}_{1}$, ${A}_{4}$, ${A}_{5}$, ${A}_{6}$, ${RA}_{1}$, ${RA}_{3}$, ${RA}_{4}$, ${RA}_{5}$, ${RA}_{9}$,${RA}_{10}$	${A}_{2}$, ${A}_{3}$, ${A}_{7}$, ${A}_{8}$, ${A}_{9}$, ${RA}_{2}$, ${RA}_{3}$, ${RA}_{6}$, ${RA}_{7}$,${RA}_{8}$
^19^	2023	${T}_{3}$, ${T}_{4}$	${A}_{2}$, ${A}_{3}$, ${A}_{4}$, ${A}_{5}$ , ${RA}_{2}$, ${RA}_{3}$, ${RA}_{5}$, ${RA}_{6}$, ${RA}_{7}$, ${RA}_{8}$, ${RA}_{9}$,${RA}_{10}$	${A}_{1}$, ${A}_{6}$, ${A}_{7}$, ${A}_{8}$, ${A}_{9}$, ${RA}_{1}$,${RA}_{4}$
^22^	2024	${T}_{4}$, ${T}_{5}$	${A}_{2}$, ${A}_{3}$, ${A}_{4}$, ${RA}_{1}$, ${RA}_{2}$, ${RA}_{3}$, ${RA}_{5}$, ${RA}_{6}$, ${RA}_{7}$, ${RA}_{8}$, ${RA}_{9}$,${RA}_{10}$	${A}_{1}$, ${A}_{5}$, ${A}_{6}$, ${A}_{7}$, ${A}_{8}$,${A}_{9}$
^23^	2024	${T}_{3}$, ${T}_{4}$, ${T}_{5}$,${T}_{7}$	${A}_{2}$, ${A}_{3}$, ${A}_{4}$, ${A}_{5}$, ${RA}_{1}$, ${RA}_{2}$, ${RA}_{3}$, ${RA}_{5}$, ${RA}_{6}$, ${RA}_{7}$, ${RA}_{8}$, ${RA}_{9}$,${RA}_{10}$	${A}_{1}$, ${A}_{6}$, ${A}_{7}$, ${A}_{8}$,${A}_{9}$

Techniques: ${T}_{1}$. Chaotic maps; ${T}_{2}$. Modular exponentiation; ${T}_{3}$. Symmetric encryption; ${T}_{4}$. Fuzzy extractor; ${T}_{5}$. PUF; ${T}_{6}$. ECC; ${T}_{7}$. Blockchain; ${T}_{8}$. Bilinear map.

Advantages: Achieve Security Properties: ${A}_{1}$. Perfect forward secrecy; ${A}_{2}$. Anonymity and Unlinkability; ${A}_{3}$. Lightweight; ${A}_{4}$. Mutual authentication; ${A}_{5}$. Session key secrecy; ${A}_{6}$. N-factor security; ${A}_{7}$. Decentralization; ${A}_{8}$. Scalability; ${A}_{9}$. Cross-cluster. and Resist Attacks: ${RA}_{1}$. Privileged-insider attack; ${RA}_{2}$. Off-line password guessing attack; ${RA}_{3}$. Impersonation attack; ${RA}_{4}$. Replay attack; ${RA}_{5}$. Man-in-middle attack; ${RA}_{6}$. Smart card (Device) loss attack; ${RA}_{7}$. Node captured attack; ${RA}_{8}$. Stolen-verifier attack; ${RA}_{9}$. Desynchronization attack; ${RA}_{10}$. Known session key attack.

Limitations: cannot achieve above security properties and may suffer from above attacks.

We noticed that in the above research on applying blockchain to healthcare, the mainstream method is to treat blockchain as the carrier of records to achieve decentralization, traceability, and prevent records from being tampered with. However, they suffer from some defects, such as incomplete decentralization, privacy disclosure, and restricted communication range. To overcome these issues, how to apply blockchain and smart contracts to design an authentication protocol for IoMT’s healthcare is a challenge.

### Motivations and contributions

IoMT, facilitated by advancements in wearable devices, IoT, and mobile internet, offers a promising solution by creating an interconnected, efficient, and personalized healthcare system. Despite its benefits, IoMT-based healthcare faces significant security challenges, including privacy risks and system vulnerabilities, which must be addressed to ensure patient and data security.

The current authentication protocols for IoMT-based healthcare often rely on Trusted Third Parties (TTP), which limits their communication range and scalability while integrating privileged parties poses a threat to privacy. The mismatch between existing research and practical application requirements motivates us to apply blockchain and smart contracts in the medical field, aiming to achieve a more effective decentralized authentication mechanism without relying on TTP. To overcome the limitations of existing authentication protocols, such as privacy disclosure, untraceability of malicious behavior, attack threats, limited communication scope, and lack of cross-cluster access, we propose a blockchain-based cross-cluster authentication protocol. Our protocol leverages the decentralized and secure nature of blockchain and smart contracts to provide efficient, secure, and scalable authentication without the need for a central authority, making it a robust solution for nowadays healthcare requirements. Our contributions are summarized as follows:

1. We propose a blockchain-based cross-cluster authentication protocol with three-factor secrecy for IoMT healthcare, which addresses several critical issues including privacy disclosure, untraceability of malicious behavior, attack threats, limited communication scope, and the lack of cross-cluster access. Our protocol leverages the decentralized nature of blockchain and the security provided by three-factor authentication to ensure robust protection of sensitive medical data while enabling seamless communication across different healthcare clusters.
2. We design a Double Anonymity Strategy (DAS) to protect user privacy and track the identity of malicious users. This strategy takes advantage of blockchain’s inherent transparency and immutability, utilizing smart contracts to replace the traditional Trusted Third Party (TTP). By doing so, we achieve efficient and automatic identity authentication, as well as secure cross-cluster communication and data transmission. The DAS ensures that user identities remain anonymous during regular operations, while still allowing for the identification of malicious actors when necessary.
3. We formally prove the security of the proposed scheme under the random oracle model, providing a rigorous theoretical foundation for its reliability. Additionally, we simulate the proposed protocol and compare it with other related protocols. The results demonstrate that our scheme not only meets the required security standards but also maintains higher efficiency. This is evidenced by its lower computational overhead and faster processing times, making it a practical and effective solution for modern IoMT healthcare systems.

In the following two sections, we present the system and adversary models, along with the preliminary concepts. Our proposed scheme is detailed in “Proposed scheme” section IV. Subsequently, “Formal security proof” section provides a formal proof, while “Informal security analysis” section offers an analysis of additional security properties. The simulation results and a comparative study with relevant schemes are presented in “Performance comparison” section. This paper is concluded in “Conclusion” section.

## System model and adversary model

### System model

In our scheme, we use the alliance blockchain instead of the public blockchain^27^. The openness of the alliance blockchain is weaker than that of the public blockchain, the participant nodes of the alliance blockchain are screened out in advance or directly designated. We assume that each hospital acts as a node and is official and trusted to jointly maintain the alliance blockchain. Each hospital has its independent registration center and smart contracts, for its convenience to verify the identity of doctors and patients at the registration stage. We require that only the smart contracts have the permission to write on the blockchain and participants can only get reading permission by joining the system. Considering the possible node failure in the network, we use the Rotation Practical Byzantine Fault Tolerance (RPBFT) consensus algorithm to improve the robustness of the system. RPBFT maintains lower time and computation overhead, keeping its operation at millisecond level. In addition, the algorithm and network complexity are independent of the number of nodes.

**Fig. 1:**
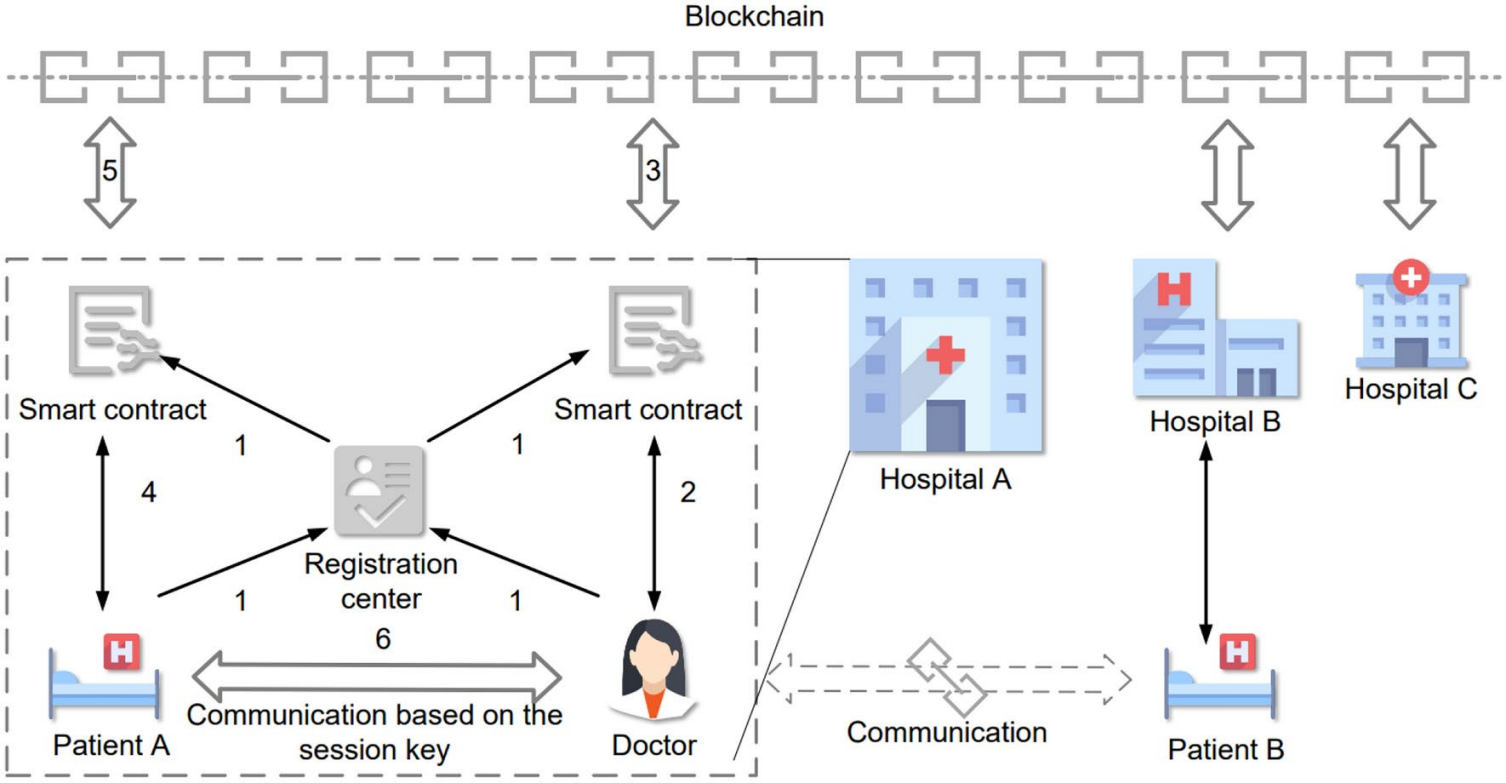
System model.

As shown in Fig. 1, many hospital nodes jointly maintain the alliance blockchain. Each node contains the patients, the doctors, the smart contracts, and the registration center. In each hospital, there are two smart contracts for users and patients respectively in private storage. The registration center is officially credible and responsible for verifying the identity of doctors and patients on registration and for disputes arbitration. The doctor and the patient register in the registration center and the smart contracts as step 1. When the doctor tries to check the patient’s status or communication, he/she sends the request to the doctors’ smart contract. The smart contract first verifies the legitimacy of the doctor after receiving the request as step 2, then uploads the parameters sent by the doctor to the blockchain and informs the patient’s device to download as step 3. Meanwhile, the smart contract updates the temporary identity of the doctor. The patient device downloads the parameters of key agreement, then calculates the session key and sends the response to the patients’ smart contract as step 4. After verifying the identity of the patient, the smart contract uploads the parameters in step 5. The doctor downloads the parameters, computes and verifies the session key. If the verification is passed, the authentication and key agreement are completed, then the doctor and the patient communicate based on the session key as step 6. Doctors can also establish communication with patients in other clusters (hospitals) in the same process. At the end of each session, the doctor’s device will automatically send the session data encrypted by the session key to the smart contract, which will upload it to the blockchain for record.

**Fig. 2:**
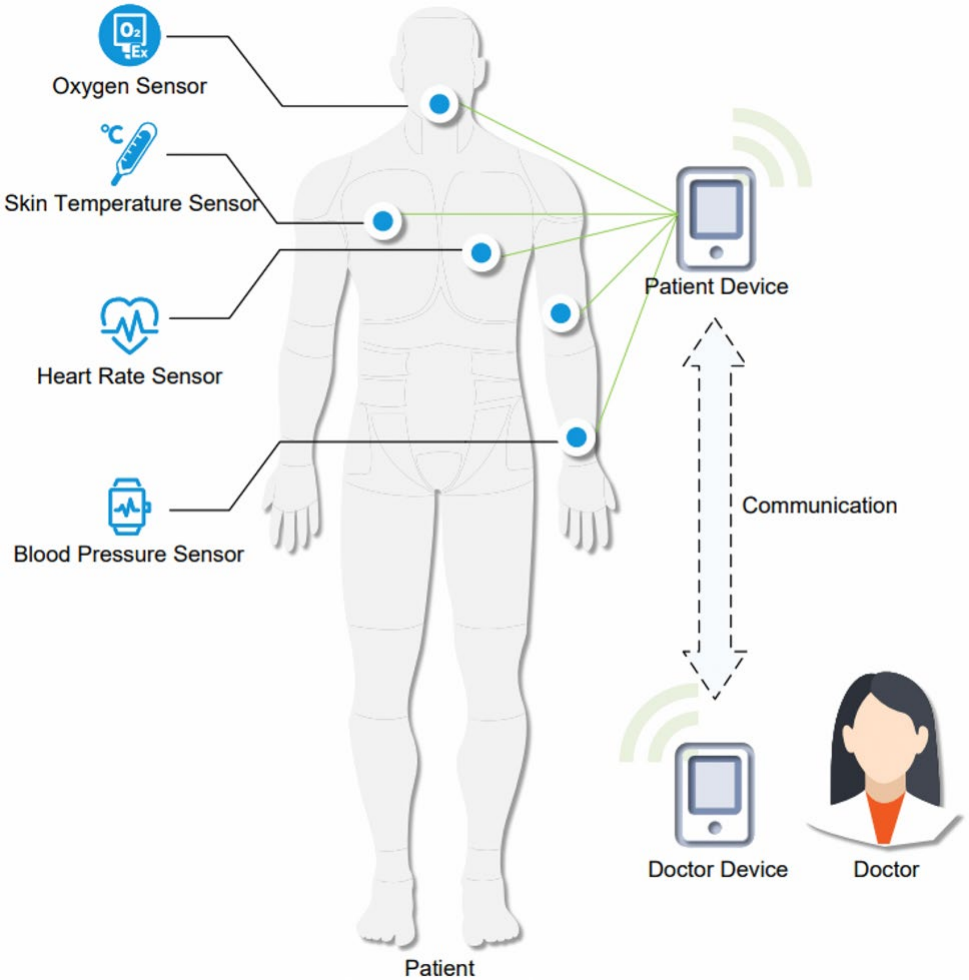
Patient sensors and device.

Figure 2 shows the relationship between the patient’s sensors and the doctor’s device. The registered patient device is connected with sensors to monitor and analyze the patient’s status in real time. If the above data exceeds the alarm threshold, the patient’s device immediately broadcasts alarms. In addition, the certified doctor can establish communication with the patient through the process shown in Fig. 1, so as to monitor the patient’s condition and view the health data stored in the patient’s device.

### Adversary model

According to the blockchain-based cross-hospital authentication scenario, and inspired by the Dolev-Yao (DY) model, the adversary model is articulated as follows:

1. The adversary, denoted as $A$, may be a legitimately registered user or an internal adversary with access to certain system components.
2. $A$ can obtain at most two of the three factors (1. user’s password; 2. biometric information; 3. information stored in the user’s devices) to launch any attacks, but cannot simultaneously obtain all three factors.
3. In the context of perfect forward secrecy, $A$ may have access to long-term private keys. In the model of three-factor secrecy, $A$ may know the user’s biometric information. In the model of known session key secrecy, $A$ may know a session key.
4. The smart contracts and registration center are trusted, but $A$ may attempt to exploit any vulnerabilities in the implementation or usage of these components.
5. This adversary model is based on the Dolev-Yao model, which assumes that the adversary has complete control over the communication channel and may impersonate, intercept, replay or modify messages to compromise the system’s security.

### Definition of the adversary in random oracle model

*Definition 1 (Participants and partnering)*: The proposed scheme $\Pi$ consists of several entities: doctors ($DOC$), smart contracts ($SC$), patient devices ($PD$), and the registration center. Among these, the doctor ($DOC$), smart contracts ($SC$), and patient’s device ($PD$) can engage in mutual authentication and session key agreement. These entities are collectively referred to as participants $P$. In the *i*-th instance, the participants $P$, $DOC$, $SC$, and $PD$ are denoted as ${I}_{P}^{i}$, ${I}_{DOC}^{i}$, ${I}_{SC}^{i}$, and ${I}_{PD}^{i}$, respectively.

*Session Identity:* In the *i*-th instance, participants that share the same session identity $SI{D}_{P}^{i}$ are considered to be in the same session. The session identity ensures that the communication and session management are correctly aligned among the entities involved.

*Partner Identity:* $PI{D}_{DOC}^{i}$ (resp. $PI{D}_{PD}^{i}$) is the partner identity of ${I}_{DOC}^{i}$ (resp. ${I}_{PD}^{i}$). This means that the partner identity records which participant each instance is interacting with or partnered with during the session.

*Acceptance State:* If a participant $P$ receives a legal and correct message, its state transitions to $Accep{t}_{P}^{i}$. This acceptance state indicates that the participant has successfully validated the incoming message and can proceed with the session.

*Partnering Conditions:* ${I}_{DOC}^{i}$ and ${I}_{PD}^{i}$ can be called partners if they satisfy the following conditions:

1. Both participants are in the acceptance state.
2. The session identities match, i.e., $SI{D}_{DOC}^{i}=SI{D}_{PD}^{i}$.
3. The partner identities are reciprocal, i.e., $PI{D}_{DOC}^{i}={I}_{PD}^{i}$ and $PI{D}_{PD}^{i}={I}_{DOC}^{i}$.
4. The agreed session keys among the partners are the same, i.e., $S{K}_{DOC}^{i}=S{K}_{PD}^{i}$.

*Definition 2 (Queries):* To simulate the malicious behavior of the adversary $A$, the queries are defined as follows:

*Execute (*${I}_{P}^{i}$*):* When this query is executed, the adversary $A$ can obtain all the messages that are transmitted openly during the public communication session. This allows $A$ to eavesdrop on the messages exchanged between the participants.

*Send (*${I}_{P}^{i}$*, *$m$*):* In this query, the adversary $A$ sends a message $m$ to participant ${I}_{P}^{i}$. If the message $m$ is legal and correct according to the protocol, ${I}_{P}^{i}$ will respond appropriately to the message. If the message is not valid, ${I}_{P}^{i}$ will neglect and ignore it. This simulates the adversary’s attempt to inject messages into the communication.

*Reveal (*${I}_{DOC}^{i}$*, *${I}_{PD}^{i}$*):* If the query *Test* has not been executed and both $S{K}_{DOC}^{i}$ and $S{K}_{PD}^{i}$ have been successfully generated, executing this query will allow $A$ to obtain the session key. This query simulates the scenario where the adversary tries to learn the session key after the key exchange process has been completed.

*Corrupt (*${I}_{DOC}^{i}$*):* When the adversary $A$ executes this query, they obtain all the secret information stored on the doctor’s device. Specifically, $A$ gains access to $\{{N}_{1},{N}_{2},HP{B}_{i},{\tau }_{i}\}$. This query represents the adversary compromising the doctor’s device.

*Test (*${I}_{DOC}^{i}$*, *${I}_{PD}^{i}$*, r):* When this query is executed, it first generates a random bit $r$. Depending on the value of $r$, the behavior of the query is as follows:

- If $r=1$, and the session key has been generated, the query returns the actual session key to $A$.
- If $r=0$, the query returns a random number instead of the actual session key.
- If neither condition is met, nothing is returned.

This query is allowed to be executed at most once, simulating a challenge-response test to verify if $A$ can distinguish the real session key from a random value.

*Definition 3 (Freshness):* An instance can be regarded as fresh if it satisfies the following conditions:

1. *Corrupt Query Condition:* The Corrupt query, which allows the adversary $A$ to gain access to sensitive information such as the internal state of a participant, has been executed at most once. Additionally, the Reveal query, which allows $A$ to learn the session key, has not been executed. This condition ensures that the adversary has limited exposure to the internal workings and secrets of the participants, maintaining the integrity of the session.
2. *Accept State Condition:* Both participants involved in the instance, denoted as ${I}_{DOC}^{i}$ (the doctor’s instance) and ${I}_{PD}^{i}$​ (the patient’s device instance), must be in the Accept state. This state indicates that the participants have successfully completed the mutual authentication process and have agreed upon a session key. The freshness condition ensures that the session is valid and uncompromised at the time of assessment.

*Definition 4 (Semantic security):* Semantic security in this context refers to the inability of the adversary $A$ to distinguish the session key from a random value. The security definition is as follows:

*Guessing Bit:* The adversary $A$ generates a random bit ${r}_{guess}$ to guess the bit $r$ generated by the *Test* query. The *Test* query is a critical part of the security game, where it either returns the actual session key or a random value based on the bit $r$.

*Condition for Compromised Security:* If ${r}_{guess}=r$, it implies that the adversary has successfully guessed whether the output of the *Test* query corresponds to the actual session key or a random number. This success indicates that $A$ has enough information to distinguish between the session key and a random value, thereby compromising the semantic security of the scheme $\Pi$.

*Advantage of the Adversary:* The adversary’s advantage is quantified as: $Ad{v}_{\Pi }^{A}=\left|2\text{Pr}\left[{r}_{guess}=r\right]-1\right|=|2\text{Pr}\left[Succes{s}_{A}\right]-1|$. This formula measures how much better the adversary is at guessing $r$ compared to random guessing. If this advantage is less than a small threshold $\eta$, the scheme $\Pi$ is considered semantically secure. Here, $\eta$ represents a negligible probability, ensuring that the adversary’s advantage is minimal.

*Semantic Security Conclusion:* For the scheme $\Pi$ to be deemed semantically secure, the adversary’s advantage $Ad{v}_{\Pi }^{A}$ must be less than the small threshold $\eta$. This ensures that $A$ cannot effectively distinguish between the session key and a random value, thereby protecting the confidentiality of the session key and maintaining the overall security of the cryptographic protocol.

## Preliminaries

### Blockchain technology

Blockchain is a decentralized, immutable, and transparent distributed ledger system, first introduced in 2008 by Nakamoto as the underlying technology for cryptocurrency^28^. It ensures that information stored is tamper-proof and traceable. Blockchain can be categorized into public, consortium, and private chains^29^, each serving different purposes based on openness, decentralization, and transaction speed. Public chains, like Bitcoin, offer high decentralization but come with higher costs. Consortium and private chains are more suitable for applications that prioritize privacy, speed, and internal oversight.

A blockchain consists of a series of blocks, each containing a header and body. The Merkle tree within each block serves as a digital fingerprint of transaction data, ensuring the integrity of records^30^.

### Smart contract

Proposed by Nick Szabo in 1997, smart contracts are self-executing agreements coded into a blockchain, eliminating the need for trusted third parties^31^. These contracts are transparent, decentralized, and automatically enforce rules and agreements between parties. They are widely used in cryptocurrency transactions and can also automate identity authentication in various applications. Smart contracts improve efficiency, fairness, and security, while traditional third-party intermediaries face issues like trust, failure points, and high costs.

### Fuzzy extractor

Fuzzy extractor technology was proposed by Dodis et al.^32^ in 2004. Fuzzy extractor algorithm can convert the input biological information $bio$ into random string $\sigma$ and public information $\tau$, which can be described as $\left(\sigma , \tau \right)= Gen\left(bio\right)$. At the same time, the random string can also be recovered by using public information and similar biological information $bi{o}{\prime}$, which can be described as $\sigma =Rep\left(bi{o}{\prime},\tau \right)$. The error of $bi{o}{\prime}$ and $bio$ is within the allowable range $\varepsilon$.

Fuzzy extractor technology is widely used in IoT and IoMT authentication protocols. Biometric information used for authentication can effectively avoid potential password guessing attacks.

### Physical unclonable function

Physical Unclonable Function (PUF) is hardware security primitive, which uses the internal physical structure to uniquely identify the digital chip^33^. Because of its uniqueness and randomness, PUF is used for security authentication and key generation.

PUF exploits variances in the chip fabrication process to manifest a distinctive function aligned with the challenge and response signals. In essence, when confronted with the identical challenge, the PUF has the capacity to furnish a consistent response falling within the acceptable margin of error. In addition, it is not feasible to speculate on the output result of PUF. Due to physical differences, the responses of different chips are different for the same challenge. PUF in the hardware device cannot be tampered. When a device is subjected to corruption attacks or side-channel attacks, the physical characteristics of the chip will change, and the response of the PUF will be different.

## Proposed scheme

The notations of the proposed scheme as shown in Table 2.

**Table 2:** Notations.

Notations	Description
$I{D}_{i}$	Personal identity of ${U}_{i}$
${PW}_{i}$	Password of ${U}_{i}$
$PI{D}_{j}$	Identity of the device of patient
$SK$	Session key
$P$	Base point of elliptic curve
$DI{D}_{i}$	Temporary identity of ${U}_{i}$
$h(.)$	Hash function
$\parallel$	Concatenation
$\oplus$	XOR operation
$Rep(.)$,$Gen(.)$	Fuzzy Extractor algorithm for reproduction and generation
${fng}_{i}$	The bioinformatics of ${U}_{i}$
${\tau }_{i}$	Public parameter of Fuzzy Extractor algorithm
${\sigma }_{i}$	Biometric key of Fuzzy Extractor algorithm
${T}_{1},{T}_{2},{T}_{3}$	Timestamps
$\Delta T$	The maximum transmission delay time
${K}_{RC}$	The secret key of registration center
$PUF(.)$	Physical unclonable function (PUF)
$\left(Ch{a}_{j},Re{s}_{j}\right)$	Challenge and response for PUF
${K}_{SCD},{K}_{SCP}$	The secret parameter of smart contract for user and patient respectively

### Initialization phase

The registration center selects its secret key ${K}_{RC}$ and computes its public key ${PK}_{RC}={K}_{RC}P$. The smart contracts for the doctors and the patients select secret keys ${K}_{SCD}$ and ${K}_{SCP}$, compute their public keys ${PK}_{SCD}={K}_{SCD}P$ and ${PK}_{SCP}={K}_{SCP}P$, respectively.

### Registration phase

The registration phase consists of the doctor registration phase and the patient’s device registration phase.

### Registration phase of a doctor

**Fig. 3:**
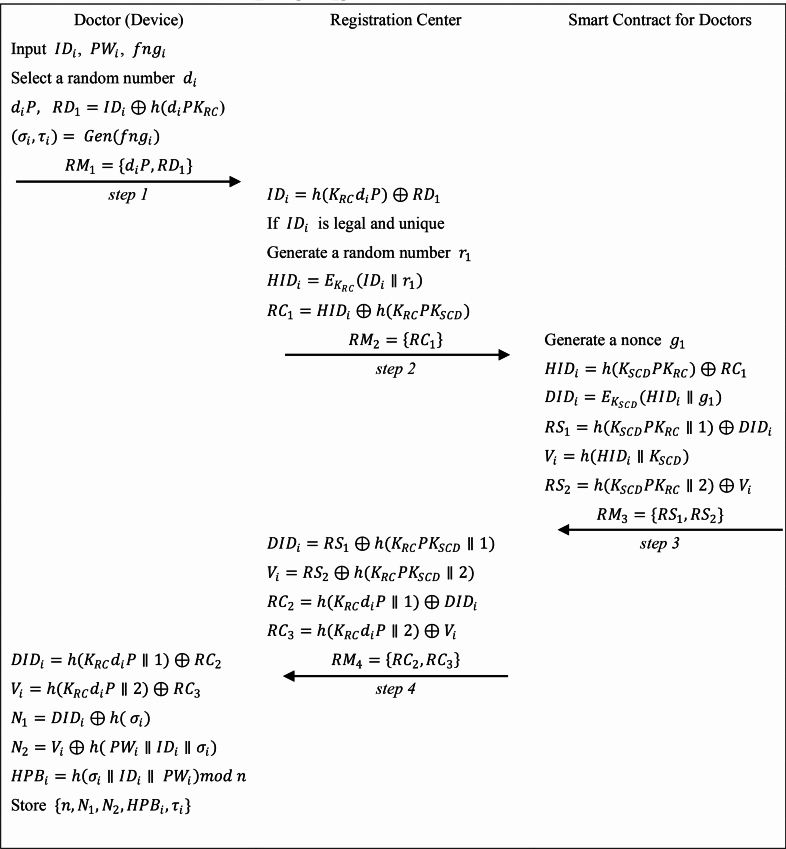
The registration phase of doctor (device).

The registration phase of the doctor is shown in Fig. 3.

**Step RU1:** The doctor inputs his/her identity $I{D}_{i}$, password $P{W}_{i}$, and biometric $fn{g}_{i}$ into the device. Then the device selects a random number ${d}_{i}$, and computes: ${d}_{i}P,$ $R{D}_{1}=I{D}_{i}\oplus h({d}_{i}{PK}_{RC}$), $({\sigma }_{i}, {\tau }_{i})= Gen({fng}_{i})$. The device transmits $R{M}_{1}=\{{d}_{i}P,R{D}_{1}\}$ to the registration center via an insecure channel.

**Step RU2:** Upon receiving $R{M}_{1}$ the registration center first computes $I{D}_{i}=h({K}_{RC}{d}_{i}P)\oplus R{D}_{1}$ and verifies the real identity and information of the doctor to ensure that it is not a malicious user counterfeiting registration. Then the registration center checks the legitimacy and uniqueness of $I{D}_{i}$, if not, requests the doctor to select a new $I{D}_{i}$. Else, the registration center generates a random number ${r}_{1}$ and computes: $HI{D}_{i}={E}_{{K}_{RC}}\left(I{D}_{i}\parallel {r}_{1}\right)$, $R{C}_{1}=HI{D}_{i}\oplus h({K}_{RC}{PK}_{SCD})$, where ${K}_{RC}$ is the secret key of the registration center. Then, the registration center transmits $R{M}_{2}=\{R{C}_{1}\}$ to the server of smart contract for doctors.

**Step RU3:** The smart contract for doctors generates a nonce ${g}_{1}$, and computes: $HI{D}_{i}=h({{K}_{SCD}PK}_{RC})\oplus R{C}_{1}$, $DI{D}_{i}={E}_{{K}_{SCD}}(HI{D}_{i}\parallel {g}_{1})$, $R{S}_{1}=h({{K}_{SCD}PK}_{RC}\parallel 1)\oplus DI{D}_{i}$, ${V}_{i}=h(HI{D}_{i}\parallel {K}_{SCD})$, ${R{S}_{2}=h({{K}_{SCD}PK}_{RC}\parallel 2)\oplus V}_{i}$. Then, it sends $R{M}_{3}=\{R{S}_{1},R{S}_{2}\}$ to the registration center. The registration center recovers $DI{D}_{i}$ and ${V}_{i}$, computes and sends $R{M}_{4}=\{R{C}_{2}=h({K}_{RC}{d}_{i}P\parallel 1)\oplus DI{D}_{i},R{C}_{3}={h({K}_{RC}{d}_{i}P\parallel 2)\oplus V}_{i}\}$ to the doctor.

**Step RU4:** On receiving $R{M}_{4}$, the doctor’s device calculates: $DI{D}_{i}=h({K}_{RC}{d}_{i}P\parallel 1)\oplus R{C}_{2}$, ${V}_{i}=h({K}_{RC}{d}_{i}P\parallel 2)\oplus R{C}_{3}$, ${N}_{1}=DI{D}_{i}\oplus h( {\sigma }_{i})$, ${N}_{2}={V}_{i}\oplus h( P{W}_{i}\parallel I{D}_{i}\parallel {\sigma }_{i})$, $HP{B}_{i}=h\left({\sigma }_{i}\parallel I{D}_{i}\parallel P{W}_{i}\right) mod n$, where* n* belongs to 16 to 256^34^, and stores $\{{n, N}_{1},{N}_{2},HP{B}_{i},{\tau }_{i}\}$.

### Registration phase of patient device

**Fig. 4:**
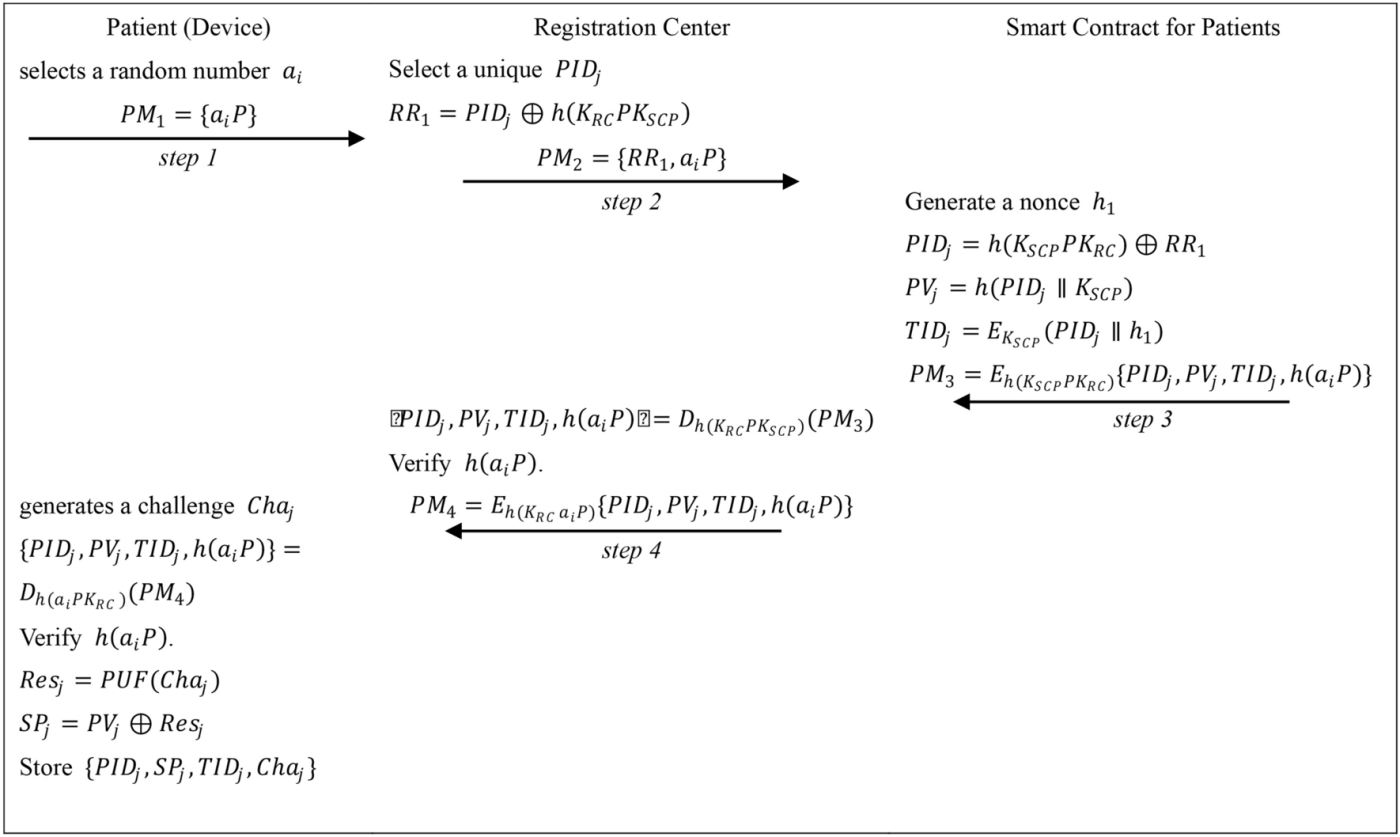
The registration phase of patient (device).

The registration phase of the patient’s device is shown in Fig. 4.

**Step RP1:** The patient’s device selects a random number ${a}_{i}$, computes and sends ${a}_{i}P$ to the registration center.

**Step RP2:** The registration center first selects a unique identity $PI{D}_{j}$ of the patient’s device, computes and sends $P{M}_{2}=\{ R{R}_{1}, {a}_{i}P\}$ to the smart contract for patients.

**Step RP3:** The smart contract generates a nonce ${h}_{1}$, and computes: $PI{D}_{j}=h({K}_{SCP}P{K}_{RC})\oplus R{R}_{1}$, $P{V}_{j}=h(PI{D}_{j}\parallel {K}_{SCP})$, $TI{D}_{j}={E}_{{K}_{SCP}}(PI{D}_{j}\parallel {h}_{1})$, ${P{M}_{3}=E}_{h({K}_{SCP}{PK}_{RC})}\{PI{D}_{j},P{V}_{j},TI{D}_{j}, h({a}_{i}P)\}$.

Then it sends $P{M}_{3}$ to the registration center. The registration center decrypts $P{M}_{3}$ by ${{h(K}_{RC}PK}_{SCP}$), and checks the correctness of $h({a}_{i}P)$. After that it computes and sends $P{M}_{4}={E}_{h({K}_{RC }{a}_{i}P)}\{PI{D}_{j},P{V}_{j},TI{D}_{j},h({a}_{i}P)\}$ to the patient’s device.

**Step RP4:** The patient’s device generates a challenge $Ch{a}_{j}$, and computes: ${(PI{D}_{j},P{V}_{j},TI{D}_{j},h({a}_{i}P))=D}_{h({a}_{i}P{K}_{RC })}\{PI{D}_{j},P{V}_{j},TI{D}_{j},h({a}_{i}P)\}$, checks the correctness of $h({a}_{i}P)$ and computes: $Re{s}_{j}=PUF(Ch{a}_{j})$, $S{P}_{j}=P{V}_{j}\oplus Re{s}_{j}$.

Where $PUF(.)$ is the physical unclonable function. The device stores $\{PI{D}_{j},S{P}_{j},TI{D}_{j},Ch{a}_{j}\}$.

### Mutual authentication and key agreement phase

**Fig. 5:**
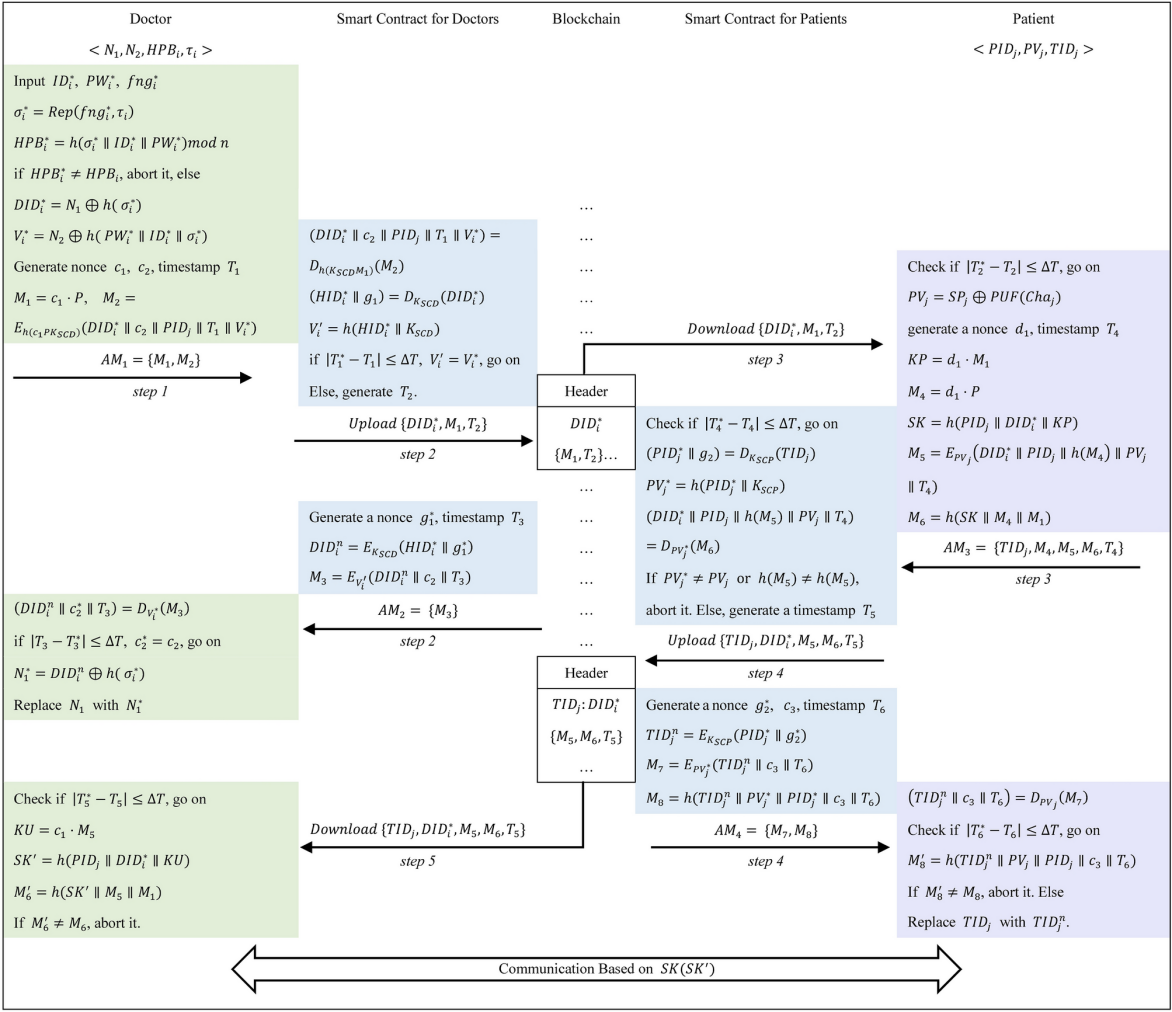
Mutual authentication and key agreement phase.

If a doctor wants to obtain a patient’s body information (his/her identity is $PI{D}_{j}$) in the same hospital or different hospitals, he/she and the patient’s device needs to pass the identity authentication of the smart contract first, and then agree on the session key through blockchain as shown in Fig. 5. The details are as follows:

**Step AK1:** The doctor first inputs identity $I{D}_{i}^{*}$, password $P{W}_{i}^{*}$, and biometric $fn{g}_{i}^{*}$ into the device, and the device computes: ${\sigma }_{i}^{*}=R\text{e}p\left(fn{g}_{i}^{*},{\tau }_{i}\right)$, $HP{B}_{i}^{*}=h\left({\sigma }_{i}^{*}\parallel I{D}_{i}^{*}\parallel P{W}_{i}^{*}\right)mod n$.

if $HP{B}_{i}^{*}\ne HP{B}_{i}$, abort it. Else, it computes: $DI{D}_{i}^{*}={N}_{1}\oplus h( {\sigma }_{i}^{*})$, ${V}_{i}^{*}={N}_{2}\oplus h\left( P{W}_{i}^{*}\parallel I{D}_{i}^{*}\parallel {\sigma }_{i}^{*}\right)$.

Then, the device generates nonce ${c}_{1}$, ${c}_{2}$ and a timestamp ${T}_{1}$, and computes: ${M}_{1}={c}_{1}\cdot P$, ${M}_{2}={E}_{h({c}_{1}P{K}_{SCD})}(DI{D}_{i}^{*}\parallel {c}_{2}\parallel PI{D}_{j}\parallel {T}_{1}\parallel {V}_{i}^{*})$. After that, the doctor’s device sends $A{M}_{1}=\{{M}_{1},{M}_{2}\}$ to the smart contract for doctors via a public channel.

**Step AK2:** The smart contract for doctors gets the current timestamp ${T}_{1}^{*}$ and computes: $(DI{D}_{i}^{*}\parallel {c}_{2}\parallel PI{D}_{j}\parallel {T}_{1}\parallel {V}_{i}^{*})={D}_{h({K}_{SCD}{M}_{1})}({M}_{2})$, $\left(HI{D}_{i}^{*}\parallel {g}_{1}\right)={D}_{{K}_{SCD}}(DI{D}_{i}^{*})$, ${V}_{i}{\prime}=h(HI{D}_{i}^{*}\parallel {K}_{SCD})$, if $\left|{T}_{1}^{*}-{T}_{1}\right|>\Delta T$ or ${V}_{i}{\prime}\ne {V}_{i}^{*}$, abort it. Else, the smart contract generates a timestamp ${T}_{2}$, uploads $\{DI{D}_{i}^{*},{M}_{1},{T}_{2}\}$ onto the blockchain, and notifies the patient’s device of $PI{D}_{j}$ to download them.

The smart contract for doctors generates a nonce ${g}_{1}^{*}$, the current timestamp ${T}_{3}$, and computes: $DI{D}_{i}^{n}={E}_{{K}_{SCD}}\left(HI{D}_{i}^{*}\parallel {g}_{1}^{*}\right)$, ${M}_{3}={E}_{{V}_{i}{\prime}}(DI{D}_{i}^{n}\parallel {c}_{2}\parallel {T}_{3})$. Then, the smart contract for doctors sends $A{M}_{2}= \{{M}_{3}\}$ to the doctor’s device.

On receiving $\{{M}_{3}\}$ from the smart contract for doctors, the doctor’s device computes: $\left(DI{D}_{i}^{n}\parallel {c}_{2}^{*}\parallel {T}_{3}\right)={D}_{{V}_{i}^{*}}\left({M}_{3}\right)$.

The device generates the current timestamp ${T}_{3}^{*}$, and checks if $\left|{T}_{3}^{*}-{T}_{3}\right|\le\Delta T$ and ${c}_{2}^{*}={c}_{2}$, if not, terminates the session, else, replaces ${N}_{1}$ with ${N}_{1}^{*}$. ${N}_{1}^{*}=DI{D}_{i}^{n}\oplus h( {\sigma }_{i}^{*})$.

**Step AK3:** The patient’s device downloads $\{DI{D}_{i}^{*},{M}_{1},{T}_{2}\}$ from the blockchain, then checks the freshness of ${T}_{2}$, if not, aborts it, else, computes: $P{V}_{j}=S{P}_{j}\oplus PUF(Ch{a}_{j})$.

The patient’s device generates a nonce ${d}_{1}$, the current timestamp ${T}_{4}$, and computes: $KP={d}_{1}\cdot {M}_{1}$, ${M}_{4}={d}_{1}\cdot P$, $SK=h(PI{D}_{j}\parallel DI{D}_{i}^{*}\parallel KP)$, ${M}_{5}={E}_{P{V}_{j}}(DI{D}_{i}^{*}\parallel PI{D}_{j}\parallel {h(M}_{4})\parallel P{V}_{j}\parallel {T}_{4})$,${M}_{6}=h(SK\parallel {M}_{4}{\parallel M}_{1})$. Then device sends $A{M}_{3}= \{TI{D}_{j},{M}_{4},{M}_{5},{M}_{6},{T}_{4}\}$ to the smart contract for patients.

**Step AK4:** Upon receiving $\{TI{D}_{j},{M}_{4},{M}_{5},{M}_{6},{T}_{4}\}$ from the patient’s device, the smart contract for patients first checks the freshness of ${T}_{4}$, if not, terminates the session, else, it computes: $(PI{D}_{j}^{*}\parallel {g}_{2})={D}_{{K}_{SCP}}(TI{D}_{j})$, $P{V}_{j}^{*}=h(PI{D}_{j}^{*}\parallel {K}_{SCP})$, ($DI{D}_{i}^{*}\parallel PI{D}_{j}\parallel {h(M}_{5})\parallel P{V}_{j}\parallel {T}_{4}$) = ${D}_{P{V}_{j}^{*}}$(${M}_{6}$). If $P{V}_{j}^{*}\ne P{V}_{j}$ or ${h(M}_{5})\ne {h(M}_{5})$, the smart contract aborts the session, else, generates a timestamp ${T}_{5}$, and uploads $\{TI{D}_{j},DI{D}_{i}^{*},{M}_{5},{M}_{6},{T}_{5}\}$ on the blockchain. Then, the smart contract notifies the doctor’s device to download them.

The smart contract generates nonce ${g}_{2}^{*}$, ${c}_{3}$, timestamp ${T}_{6}$, and computes: $TI{D}_{j}^{n}={E}_{{K}_{SCP}}(PI{D}_{j}^{*}\parallel {g}_{2}^{*})$, ${M}_{7}={E}_{P{V}_{j}^{*}}(TI{D}_{j}^{n}\parallel {c}_{3}\parallel {T}_{6})$, ${M}_{8}=h(TI{D}_{j}^{n}\parallel P{V}_{j}^{*}\parallel PI{D}_{j}^{*}\parallel {c}_{3}\parallel {T}_{6})$. Then it sends $A{M}_{4}= \{{M}_{7},{M}_{8}\}$ to the patient’s device.

The patient’s device computes: $\left(TI{D}_{j}^{n}\parallel {c}_{3}\parallel {T}_{6}\right)={D}_{P{V}_{j}}\left({M}_{7}\right)$. The device checks the freshness of ${T}_{6}$, if not, terminates it. Else, computes: ${M}_{8}{\prime}=h(TI{D}_{j}^{n}\parallel P{V}_{j}\parallel PI{D}_{j}\parallel {c}_{3}\parallel {T}_{6})$. If ${M}_{8}{\prime}\ne {M}_{8}$, abort it. Else, replace $TI{D}_{j}$ with $TI{D}_{j}^{n}$.

**Step AK5:** After downloading $\{TI{D}_{j},DI{D}_{i}^{*},{M}_{5},{M}_{6},{T}_{5}\}$ from the blockchain, the doctor’s device first checks the freshness of ${T}_{5}$, if not, terminates the session, else, it computes: $KU={c}_{1}\cdot {M}_{5}$, $S{K}{\prime}=h(PI{D}_{j}\parallel DI{D}_{i}^{*}\parallel KU)$, ${M}_{6}{\prime}=h(S{K}{\prime}\parallel {M}_{5}{\parallel M}_{1})$.

If ${M}_{6}{\prime}\ne {M}_{6}$, the device aborts the session, otherwise, the authentication and session key agreement are successfully completed.

### Anonymous identity tracking

Although the identities of the doctor and the patient’s device are dynamically anonymous, their true identities can still be tracked when needed.

In case of dispute, the patient can submit the doctor’s $DI{D}_{i}$ to the smart contract for doctors, who can recover the doctor’s pseudo-identity $HI{D}_{i}$ by calculating $\left(HI{D}_{i}\parallel {g}_{1}\right)={D}_{{K}_{SCD}}(DI{D}_{i})$, where $DI{D}_{i}$ is the doctor’s anonymous identity, ${K}_{SCD}$ is the secret parameter of the smart contract, ${g}_{1}$ is a nonce, and $HI{D}_{i}={E}_{{K}_{RC}}\left(I{D}_{i}\parallel {r}_{1}\right)$. After that, the smart contract for doctors sends $HI{D}_{i}$ to the registration center, who can recover the doctor’s real identity $I{D}_{i}$ by computing $\left(I{D}_{i}\parallel {r}_{1}\right)={D}_{{K}_{RC}}(HI{D}_{i})$, where ${K}_{RC}$ is the registration center’s secret parameter, ${r}_{1}$ is a nonce.

The smart contract for patients recovers the identity of the patient’s device by calculating $(PI{D}_{j}\parallel {h}_{1})={D}_{{K}_{SCP}}(TI{D}_{j})$, where $PI{D}_{j}$ is the identity of the device, ${h}_{1}$ is a nonce, $TI{D}_{j}$ is the device’s anonymous identity, and ${K}_{SCP}$ is the secret parameter of the smart contract.

## Formal security proof

In this segment, we furnish a formal proof of security within the context of the random oracle model, validating the semantic security of the proposed blockchain-based scheme $\Pi$. We aim to illustrate that the advantage of an adversary $A$ in breaking the semantic security of $\Pi$ within probabilistic polynomial time (PPT) is negligible, under the assumption of the intractability of ECDLP.

### Formal security proof

#### Theorem 1

*We define the advantage of breaking the semantic security of* $\Pi$ *by* $A$ *in PPT is* $Ad{v}_{\Pi }^{A}$ *.* $A$ *runs* ${q}_{hash}$ *,* ${q}_{send}$ *, and* ${q}_{exe}$ *times Hash, Send, and Execute queries, respectively.* ${l}_{hash}$ *,* ${l}_{nonce}$ *, and* ${l}_{\sigma }$ *represent the bit-length of the hash, nonce, and the biometric key.* ${C}{\prime}$ *and* ${s}{\prime}$ *are the regression parameters of the password space. The advantage can be defined as:*

$$Ad{v}_{\Pi }^{A}\le \frac{{q}_{hash}^{2}}{{2}^{{l}_{hash}}}+\frac{({q}_{exe}+{q}_{send}{)}^{2}}{{2}^{{l}_{nonce}-1}}+{C}^{{{\prime}}}\cdot {q}_{send}^{{s}^{{{\prime}}}}\cdot \frac{{q}_{send}}{{2}^{{l}_{\sigma }-1}}+2{q}_{hash}\cdot \left(Ad{v}_{A}^{ECDLP}+\frac{1}{{2}^{{l}_{hash}}}\right).$$

#### *Proof*

We define a series of games $Gam{e}_{i}$ to simulate attacks against semantic security launched by the adversary $A$. $Succes{s}_{i}$ represents $A$ breaks the semantic security of $\Pi$ in $Gam{e}_{i}$. The definitions are as follows:

$Gam{e}_{0}$*:* This constitutes a genuine attack initiated by the adversary $A$. She/he first selects a random bit ${r}_{guess}$. According to the definition, we have:

1 $$\begin{array}{c}Ad{v}_{\Pi }^{A}=|2\text{Pr}\left[Succes{s}_{0}\right]-1|\end{array}$$

$Gam{e}_{1}$*:* In this simulation, the adversary $A$ initiates the *Execute* query to acquire openly transmitted messages between participants. Finally, $A$ executes the *Test* query and judges whether the output of *Test* is the actual session key.

In the proposed scheme, the session key is defined as $SK=h(PI{D}_{j}\parallel DI{D}_{i}^{*}\parallel {d}_{1}\cdot {c}_{1}\cdot P)$. Among the intercepted messages, parameters $DI{D}_{i}^{*}$, ${M}_{1}$, $PI{D}_{j}$, and ${M}_{5}$​ are related to the session key. However, the adversary $A$ cannot establish an association between these messages and the session key due to the Computational Diffie-Hellman Problem (CDHP). Even though $A$ intercepts all the transcripts, whether the result corresponds to the session key remains indistinguishable.

Based on the above analysis, we get:

2 $$\begin{array}{c}\text{Pr}\left[Succes{s}_{1}\right]=\text{Pr}\left[Succes{s}_{0}\right]\end{array}$$

$Gam{e}_{2}$*:* The adversary $A$ tries to find collisions in the hash values used in the protocol. According to the birthday paradox, which describes the probability of two or more values hashing to the same output, the maximum probability of a hash collision is $\frac{{q}_{hash}^{2}}{{2}^{{l}_{hash}+1}}$​​, where ${q}_{hash}$​ is the number of hash queries and ${l}_{hash}$ is the length of the hash output in bits. Since hash functions are designed to be collision-resistant, the likelihood of $A$ successfully finding a collision is extremely low if ${l}_{hash}$​ is large enough (e.g., 256 bits). Thus, $A$ cannot easily exploit hash collisions to distinguish the session key. Nonces (numbers used once) are crucial for ensuring the freshness of messages. The collision probability of a nonce is at most $\frac{({q}_{exe}+{q}_{send}{)}^{2}}{{2}^{{l}_{nonce}}}$​, where ${q}_{exe}$​ and ${q}_{send}$ are the number of *Execute* and *Send* queries, respectively, and ${l}_{nonce}$​ is the bit length of the nonce. Nonce collisions are similarly unlikely if ${l}_{nonce}$​ is sufficiently large. Nonce collisions could undermine the security of the protocol by allowing $A$ to replay or predict messages. However, a properly sized nonce (e.g., 256 bits) minimizes this risk.

XOR operations are used in various cryptographic protocols to combine different values. The security of these operations relies on the unpredictability of the inputs. Even if $A$ intercepts XOR values, without knowing the individual components, it remains computationally infeasible to reverse-engineer them. The strength of the XOR operation in this context depends on the secrecy and randomness of the involved values (e.g., session keys, nonces). Given the above analysis, the probability that $A$ can successfully distinguish the session key in Game 2 by exploiting hash collisions, nonce collisions, or XOR operations is negligible. Thus, the probability difference between the success in Game 2 and Game 1 is bounded by:

3 $$\begin{array}{c}\text{Pr}\left[Succes{s}_{2}\right]-\text{Pr}\left[Succes{s}_{1}\right]\le \frac{{q}_{hash}^{2}}{{2}^{{l}_{hash}+1}}+\frac{({q}_{exe}+{q}_{send}{)}^{2}}{{2}^{{l}_{nonce}}}\\ \end{array}$$

$Gam{e}_{3}$*:* Which simulates the corruption attack based on the doctor’s device launched by $A$. On beginning, $A$ executes *Execute* to obtain $\{{N}_{1},{N}_{2},HP{B}_{i},{\tau }_{i}\}$, where ${N}_{1}=DI{D}_{i}\oplus h({\sigma }_{i})$, ${N}_{2}={V}_{i}\oplus h( P{W}_{i}\parallel I{D}_{i}\parallel {\sigma }_{i})$, and $HP{B}_{i}=h\left({\sigma }_{i}\parallel I{D}_{i}\parallel P{W}_{i}\right)mod n$, ${\tau }_{i}$ is the public parameter for recovering the biometric key ${\sigma }_{i}$ by calculating ${\sigma }_{i}=R\text{e}p\left(fn{g}_{i},{\tau }_{i}\right)$. The adversary $A$ needs to guess the password $P{W}_{i}$ and the biometric key ${\sigma }_{i}$ to obtain valuable information and launch attacks. According to Zipf’s law^34^, which describes the frequency distribution of words (or passwords) in natural language, the maximum probability of guessing a password is ${C}{\prime}\cdot {q}_{send}^{{s}{\prime}}$​. The password dictionary space is denoted as $|D|$, where ${C}{\prime}$ and ${s}{\prime}$ are the regression parameters of $|D|$. The probability of guessing ${\sigma }_{i}$ is at most $\frac{{q}_{send}}{{2}^{{l}_{\sigma }}}$​​, where ${l}_{\sigma }$ represents the bit length of the biometric key ${\sigma }_{i}$​. This bound illustrates that the probability of a successful corruption attack by $A$ remains limited, ensuring that the proposed scheme’s security is maintained even when $A$ attempts to exploit potential weaknesses through corruption attacks. Therefore, we get:

4 $$\begin{array}{c}\text{Pr}\left[Succes{s}_{3}\right]-\text{Pr}\left[Succes{s}_{2}\right]\le {C}{\prime}\cdot {q}_{send}^{{s}{\prime}}\cdot \frac{{q}_{send}}{{2}^{{l}_{\sigma }}}\end{array}$$

$Gam{e}_{4}$*:* In the proposed scheme, the session key $SK=h(PI{D}_{j}\parallel DI{D}_{i}^{*}\parallel {d}_{1}\cdot {c}_{1}\cdot P)$, whose agreement and generation are based on CDHP and one-way hash. This game simulates that $A$ tends to calculate the session key after executing queries *Execute* and *Hash*. In the first scenario, $A$ launches a hash collision attack against the session key. The probability of a hash collision occurring is at most $\frac{{q}_{hash}}{{2}^{{l}_{hash}}}$​​, where ${q}_{hash}$​ is the number of hash queries made by $A$ and ${l}_{hash}$ is the bit length of the hash output. The likelihood of $A$ successfully finding a hash collision is inversely proportional to the bit length of the hash output. In the second scenario, $A$ obtains ${M}_{1}={c}_{1}\cdot P$ and ${M}_{5}={d}_{1}\cdot P$ and then attempts to calculate ${c}_{1}\cdot {d}_{1}\cdot P$. The maximum probability of success for this calculation is ${q}_{hash}\cdot Ad{v}_{A}^{ECDLP}$​, where $Ad{v}_{A}^{ECDLP}$ represents $A$ 's advantage in solving the Elliptic Curve Discrete Logarithm Problem (ECDLP). According to the definition, $Ad{v}_{A}^{ECDLP}<\eta$, where $\eta$ is a sufficiently small constant representing the negligible advantage of $A$ in solving the ECDLP.

Combining both scenarios, the probability difference between the success in Game 4 and Game 3 is bounded by:

5 $$\begin{array}{c}\\ \text{Pr}\left[Succes{s}_{4}\right]-\text{Pr}\left[Succes{s}_{3}\right]\le {q}_{hash}\cdot \left(Ad{v}_{A}^{ECDLP}+\frac{1}{{2}^{{l}_{hash}}}\right)\end{array}$$

From the above games, $A$ still have no advantage to guess the random bit $r$. That is:

6 $$\begin{array}{c}\text{Pr}\left[Succes{s}_{4}\right]=\frac{1}{2}\end{array}$$

From formula (1), we know:

7 $$\begin{array}{c}\frac{1}{2}Ad{v}_{\Pi }^{A}=\left|\text{Pr}\left[Succes{s}_{0}\right]-\frac{1}{2}\right|\end{array}$$

Therefore, combining formulas (2) to (7), we get:

$$\begin{aligned}\frac{1}{2}Ad{v}_{\Pi }^{A} & =\left|\text{Pr}\left[Succes{s}_{1}\right]-\text{Pr}\left[Succes{s}_{4}\right]\right|\le \left|\text{Pr}\left[Succes{s}_{1}\right]-\text{Pr}\left[Succes{s}_{2}\right]\right| \\ & \quad +\left|\text{Pr}\left[Succes{s}_{2}\right]-\text{Pr}\left[Succes{s}_{3}\right]\right|+\left|\text{Pr}\left[Succes{s}_{3}\right]-\text{Pr}\left[Succes{s}_{4}\right]\right|\end{aligned}$$

That is: $$Ad{v}_{\Pi }^{A}\le \frac{{q}_{hash}^{2}}{{2}^{{l}_{hash}}}+\frac{({q}_{exe}+{q}_{send}{)}^{2}}{{2}^{{l}_{nonce}-1}}+{C}{\prime}\cdot {q}_{send}^{{s}{\prime}}\cdot \frac{{q}_{send}}{{2}^{{l}_{\sigma }-1}}+2{q}_{hash}\cdot \left(Ad{v}_{A}^{ECDLP}+\frac{1}{{2}^{{l}_{hash}}}\right)$$.

## Informal security analysis

### Stolen-verifier attack

In our proposed system, both the smart contracts and the registration center are considered trusted entities and do not retain verification tables for identity authentication. This design aspect enhances the scheme’s resilience against stolen-verifier attacks.

### Desynchronization attack

The temporary user identity $DI{D}_{i}$ will be changed in each session and only the user (doctor) stores $DI{D}_{i}$, where $DI{D}_{i}={E}_{{K}_{SCD}}(HI{D}_{i}\parallel {g}_{1})$. Even if the adversary intercepts, tampers, or deletes the message $A{M}_{2}$ to prevent the user (doctor) from updating the temporary identity, the user (doctor) can still successfully complete the next session of authentication and session key agreement with the previous temporary identity. This strategy of updating user temporary identity avoids desynchronized updates. Similarly, the desynchronization attack against the patient device does not work. Therefore, our scheme can resist desynchronization attacks.

### Device node captured attack

The device of the patient stores $<PI{D}_{j},S{P}_{j},TI{D}_{j},Ch{a}_{j}>$, where $S{P}_{j}=P{V}_{j}\oplus PUF(Ch{a}_{j})$, $TI{D}_{j}={E}_{{K}_{SCP}}(PI{D}_{j}\parallel {h}_{1})$. According to the characteristics of PUF, even if the adversary obtains the device, he/she cannot recover the secret parameters $P{V}_{j}$ by side-channel attacks and corruption attacks. In addition, the adversary cannot obtain the secret parameter ${K}_{SCP}$ and patient’s real identity.

### Off-line identity/password guessing attack

In the device, the identity $I{D}_{i}$ and the password $P{W}_{i}$ are included in $HP{B}_{i}=h\left({\sigma }_{i}\parallel I{D}_{i}\parallel P{W}_{i}\right) mod n$, assume an adversary compromised the device and obtained the biometric information, and attempt to guess $(I{D}_{i}\parallel P{W}_{i})$ to match $HP{B}_{i}$. When $n=256$, there are $\left|{D}_{PW}\right|*\left|{D}_{ID}\right|/n\approx {2}^{32}$ candidates of $(I{D}_{i}\parallel P{W}_{i})$ pair. Therefore, the adversary cannot know which pair is correct, the proposed scheme can resist off-line identity/password guessing attacks.

### Three-factor secrecy

Assume an adversary compromised the device and obtained the biometric information, according to above analysis, he/she cannot verify the guessed $(I{D}_{i}\parallel P{W}_{i})$ pair.

Assuming an adversary has acquired the biometric information and password, he/she remain unaware of the stored information $\{{n, N}_{1},{N}_{2},HP{B}_{i},{\tau }_{i}\}$ so he/she cannot launch any attacks.

Assume an adversary obtained the password and compromised the device, he/she cannot obtain any valuable information to launch any attacks.

Therefore, our scheme can achieve three-factor secrecy.

### Anonymity and unlinkability

The temporary identities $DI{D}_{i}$ and $TI{D}_{j}$ are updated in each session, where $DI{D}_{i}={E}_{{K}_{SCD}}(HI{D}_{i}\parallel {g}_{1})$, $TI{D}_{j}={E}_{{K}_{SCP}}(PI{D}_{j}\parallel {h}_{1})$. The updated temporary identities are $DI{D}_{i}^{n}={E}_{{K}_{SCD}}\left(HI{D}_{i}^{*}\parallel {g}_{1}^{*}\right)$, $TI{D}_{j}^{n}={E}_{{K}_{SCP}}(PI{D}_{j}^{*}\parallel {g}_{2}^{*})$. The nonce is independent in each session run, so the different temporary identities are unlinkable. The adversary also cannot recover the real identities from the temporary identities without knowing the secret keys of the registration center and the smart contracts.

### Smart card (device of doctor) lost attack

The device of the user (doctor) stores $<{N}_{1},{N}_{2},HP{B}_{i},{\tau }_{i}>$, where ${N}_{1}=DI{D}_{i}\oplus h( {\sigma }_{i})$, ${N}_{2}={V}_{i}\oplus h( P{W}_{i}\parallel I{D}_{i}\parallel {\sigma }_{i})$, $HP{B}_{i}=h\left({\sigma }_{i}\parallel I{D}_{i}\parallel P{W}_{i}\right) mod n$, and ${\tau }_{i}$ is the public parameter of fuzzy extractor for recovering the biometric key ${\sigma }_{i}$ of the user. Suppose the adversary gets the device and obtains the information stored, he/she cannot recover any unencrypted information without the bioinformatics ${fng}_{i}$ and the password ${PW}_{i}$ of the user (doctor). Hence, in the event of the doctor’s device being lost, the adversary is unable to acquire any valuable information or execute attacks.

### Impersonation attack

Suppose the adversary attempts to forge the message $A{M}_{1}=\{{M}_{1},{M}_{2}\}$ to impersonate the doctor, where ${M}_{2}={E}_{{h(c}_{1}P{K}_{SCD})}(DI{D}_{i}^{*}\parallel {c}_{2}\parallel PI{D}_{j}\parallel {T}_{1}\parallel {V}_{i}^{*})$. However, forging ${M}_{2}$ is impossible because ${V}_{i}^{*}$ is unavailable for the adversary. Meanwhile, replaying ${M}_{2}$ is also useless because of the timestamp ${T}_{1}$ and the nonce ${c}_{2}$. Therefore, the user cannot be impersonated by the adversary.

Assume that the adversary impersonates the smart contract and forges $A{M}_{2}= \{{M}_{3}\}$ or $A{M}_{4}= \{{M}_{7},{M}_{8}\}$, where ${M}_{3}={E}_{{V}_{i}{\prime}}(DI{D}_{i}^{n}\parallel {c}_{2}\parallel {T}_{3})$. The forged messages cannot pass the authentication of the doctor because ${c}_{2}$ is unavailable. Similarly, ${M}_{8}$ and ${M}_{9}$ cannot be forged without knowing $P{V}_{j}$, where ${M}_{7}={E}_{P{V}_{j}^{*}}(TI{D}_{j}^{n}\parallel {c}_{3}\parallel {T}_{6})$ and ${M}_{8}=h(TI{D}_{j}^{n}\parallel P{V}_{j}^{*}\parallel PI{D}_{j}^{*}\parallel {c}_{3}\parallel {T}_{6})$. Therefore, the smart contracts cannot be impersonated.

### Replay attack

In our scheme, all transmitted messages are combined with the timestamp and random nonce. Therefore, the proposed scheme can resist replay attacks.

### Perfect forward secrecy

Assuming the adversary has acquired long-term keys, implying the capability to decrypt all transcripts from the authentication and key agreement process. However, the session key $SK=h(PI{D}_{j}\parallel DI{D}_{i}^{*}\parallel {c}_{1}\cdot {d}_{1}\cdot P)$ is computed based on the CDHP, the adversary cannot obtain the nonce ${c}_{1}$ and ${d}_{1}$ to calculate ${c}_{1}\cdot {d}_{1}\cdot P$ because they are not transmitted. As a result, even with knowledge of all the long-term keys, the adversary remains unable to compute past session keys. Thus, the proposed scheme attains impeccable forward secrecy.

### Known session key secrecy

Assuming that the current session key $SK$ is leaked to the adversary, where $SK=h(PI{D}_{j}\parallel DI{D}_{i}^{*}\parallel {c}_{1}\cdot {d}_{1}\cdot P)$. He/she cannot obtain any other valuable information according to $SK$. Meanwhile, the session keys are independent because of the nonce. Therefore, the adversary cannot discover or calculate previous or subsequent session keys. The proposed scheme has known session key secrecy.

### Session key secrecy

The session key $SK=h\left(PI{D}_{j}\parallel DI{D}_{i}^{*}\parallel {d}_{1}\cdot {c}_{1}\cdot P\right)=SK{\prime}=h(PI{D}_{j}\parallel DI{D}_{i}^{*}\parallel {c}_{1}\cdot {d}_{1}\cdot P)$, where ${d}_{1}$ and ${c}_{1}$ represent distinct random numbers. Due to the CDHP and the one-way hash function, an adversary would be unable to gain any valuable information, even if they were to acquire the session key.

## Performance comparison

In this section, we use several devices to simulate and analyze the proposed scheme. We deploy an alliance blockchain and smart contracts based on FISCO BCOS on Ubuntu 16.04 64bit, Intel (R) Core (TM) i7-1165G7 @ 2.80 GHz, where the nodes simulate different hospitals. The requests of doctors and patients are simulated on Python 3.8.6, Windows Intel (R) Core (TM) i5-6300HQ CPU @ 2.30 GHz, and the number of requests can be controlled. The consensus mechanism of FISCO BCOS is RPBFT, so it can accurately simulate our scheme. To highlight the advantages of our authentication scheme, we compare it with^7,8,18,19,22^ in different performance indicators. Among them, the comparison of security properties is shown in Table 3.

**Table 3:** Comparison of security properties.

	Attacks/properties	^7^	^8^	^18^	^19^	^22^	Ours
Security	n-factor security	–	✗	✗	✗	✗	✓
	Off-line password guessing attack	✓	✓	✗	✗	✓	✓
	Impersonation attack	✗	✗	✗	✓	✓	✓
	Replay attack	✓	✗	✓	✓	✓	✓
	Man-in-the-middle attack	✓	✗	✓	✓	✓	✓
	Smart card (device) loss attack	✓	✓	✗	✓	✓	✓
	Device node capture attack	✓	✓	✗	✓	✓	✓
	Stolen-verifier attack	✗	✗	✗	✓	✓	✓
	Desynchronization attack	✓	✓	✓	✓	✓	✓
	Mutual authentication	✗	✓	✓	✓	✓	✓
	Session key secrecy	✗	✗	✓	✓	✗	✓
	Know session key attack	✗	✓	✓	✓	✓	✓
	Perfect forward secrecy	✗	✗	✓	✗	✗	✓
Privacy	Doctor anonymity	✗	✗	✗	✓	✓	✓
	Patient anonymity	✗	✗	✗	✓	✓	✓
	Unlinkability	✗	✗	✗	✓	✓	✓
Applicability	Decentralization	✓	✗	✗	✗	✗	✓
	Scalability	✓	✗	✗	✗	✗	✓
	Cross-cluster	✓	✗	✗	✗	✗	✓

✓: Resist(attacks)/possess(properties), ✗: suffer(attacks)/no(properties).

The scheme of Yazdinejad et al.^7^ is a decentralized authentication scheme based on blockchain for hospital networks, which uses public blockchain and PoW to realize decentralization, message traceability, and cross-cluster communication. The scheme of Xiang et al.^8^ is a permissioned blockchain-based identity management and user authentication protocol for E-healthcare. In their protocol, doctors and patients are regarded as users, and the blockchain is used as a trusted distributed ledger to record user registration information.

### Computation and energy overheads

Firstly, we simulate the case that a single node completes authentication and key agreement when it receives multiple requests concurrently, and calculate the average time. This represents the situation where doctors and patients in the same hospital request to establish communication. The specific parameters of the consensus mechanism PoW are not given in^7^, we refer to the research on PoW in^35^. In the competitive consensus mechanism, calculating the random value in each round is an uncertain calculation problem, therefore, the delay of blocks generated by nodes in different rounds fluctuates from 50 $ms$ to 6000 $ms$. PoW has the characteristics of high computational overhead and low Transaction Per Second (TPS). The goal of this simulation is to verify the authentication efficiency of a single node in a single cluster, so the competition between nodes is not considered. We use ${T}_{H}$, ${T}_{ECC}$, ${T}_{SE}$, ${T}_{POW}$, and ${T}_{RPBFT}$ to denote the time overhead of Hash operation, elliptic curve multiplication, symmetric encryption, and consensus mechanism of PoW and RPBFT, respectively. The environment of Raspberry Pi 4B is quad-core 64bits ARM Cortex-A72, 1.5 GHz, and we use 2 GB LPDDR4 SDARM to simulate the portable devices of doctors and patients. In the above environment, ${T}_{H}\approx 0.018 ms$, ${T}_{ECC}\approx 2.610 ms$, ${T}_{SE}\approx 0.551 ms$. ${T}_{POW}$ and ${T}_{RPBFT}$ vary according to the number of nodes and the number of requests. Generally speaking, ${T}_{POW}\ge 142.9 ms$ and $0.01 ms\le {T}_{RPBFT}\le 1 ms$. The overhead of our scheme is mainly the computational overhead of authentication and the overhead of the consensus mechanism. We denote the overhead of a single authentication as $13{T}_{H}+6{T}_{ECC}+2{T}_{SE}+{T}_{RPBFT}$($\approx 16.996 ms$). According to the specific protocol of Xiang et al., the overhead of the protocol is denoted as $12{T}_{H}+8{T}_{ECC}$($\approx 21.096 ms$). Since Yazdinejad et al. do not propose a specific protocol, we believe that the overhead is slightly greater than ${T}_{POW}$($\ge 142.9 ms$).

In Kumar et al.’s scheme^18^, patients and doctors hold each other’s public keys and negotiate a session key through the cloud server. The session key negotiation involves multiple symmetric encryptions, signatures, and ECC point multiplications. The cost of a single authentication is approximately $26{T}_{H}+4{T}_{Sig}+6{T}_{SE}+4{T}_{ECC}$($\approx 2952.842 ms$), and it increases linearly with the number of authentication requests. In Chen et al.’s scheme^19^, users agree with a sensor node a session key through the gateway. The security of the scheme is based on pre-shared secrets and hash functions, and it does not provide forward security. Specific security details can be found in Table 3. The authentication process involves 30 hash operations and asymmetric encryption/decryption. We measured that, according to the parameters given in this paper, encrypting and decrypting 256 bits data using AES-256 takes approximately 734.657 $ms$. The cost of a single authentication is approximately $30{T}_{ECC}+2{T}_{ASE}$($\approx \text{1,469.854} ms$), and it increases linearly with the number of authentication requests. In Mahmood et al.’s scheme^22^, users and sensors negotiate a session key through the cloud server. The authentication involves $21{T}_{H}$($\approx 0.378 ms$).

**Fig. 6:**
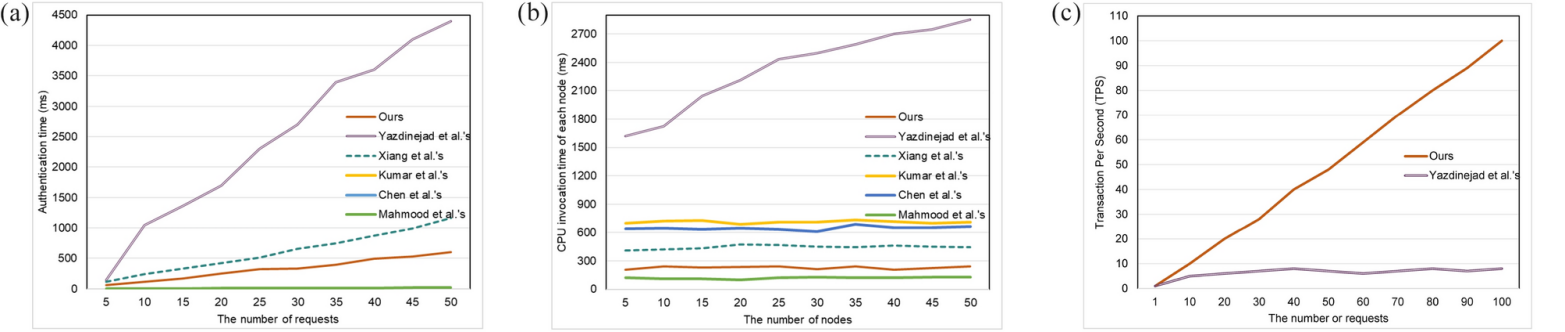
(**a**) The authentication time of one node. (**b**) CPU invocation time of each node. (**c**) The transaction per second (TPS) of blockchain.

As shown in Fig. 6a, we evaluated the time complexity of six schemes to deal with different numbers of authentication requests. We simulate the overhead spent by a single node processing different numbers of authentication requests in parallel. This reflects the efficiency of concurrently processing multiple authentications in one hospital. In our solution, the cost of processing one authentication request is about 12 ms, which can meet the requirements of fast access to medical data in emergency medical environments. we tested the blockchain’s throughput under high-concurrency conditions. On the other hand, the blockchain throughput scales with the number of requests, and the RPBFT consensus mechanism maintains a throughput of 1000 TPS to 100,000 TPS. This demonstrates that our solution can fully meet the demand for real-time, high-volume data processing in practical emergency scenarios. Additionally, the total overhead for one node to process 50 requests is approximately 500 ms, which we believe is fast enough to ensure efficient and timely data access in high-pressure environments like emergency rooms, without compromising patient care. The overhead of Xiang et al.’s protocol is slightly higher than our scheme. The inefficiency of PoW consensus mechanism limits the throughput of public blockchain, hence each authentication of Yazdinejad et al.’s protocol is accompanied by unnecessary overhead, resulting in that its authentication time is much higher than the above two schemes. In the case of a single cluster, the centralized authentication protocols are generally more efficient than the blockchain-based decentralized authentication protocols. Nevertheless, in the actual medical scenario, the centralized authentication protocols cannot meet the needs of cross-hospitals communication.

In order to test the scalability of the blockchain-based authentication protocols and the computational overhead in the case of multiple nodes, we simulated the situation by calculating the computational overhead of protocols under different numbers of nodes. We record the time spent calling the CPU during the simulation in the python environment. As shown in Fig. 6b, we tested the CPU invocation time of a single node with different numbers of nodes and each node handles 20 authentication requests. For Yazdinejad et al.’s scheme, the computational cost of nodes keeps approximately linear growth to the number of nodes. The reason for the increasing is that nodes based on the PoW consensus mechanism need to calculate a large number of hash operations to find an eigenvalue to compete for the permission of generating blocks. Besides, the consensus mechanism requires all nodes to participate in the confirmation operation, resulting in low efficiency and long confirmation time which is difficult to be reduced. The RPBFT consensus mechanism used in our scheme can replace the verification node in turn. Its algorithm complexity and network complexity are independent of the number of nodes, and its scalability and consensus efficiency are higher than Practical Byzantine Fault Tolerance (PBFT). Therefore, even with an increase in the number of nodes, the overhead of each node in our solution remains below 300 ms when processing the same number of requests, which can meet the needs of multiple medical service providers to access patient data across hospitals in real-time. According to the simulation results, the computational overhead of our scheme is much lower than the other schemes.

As shown in Fig. 6c. We test the Transaction Per Second (TPS) of the blockchain in our scheme and Yazdinejad’s scheme. In Xiang et al.’s scheme, the blockchain is only used in the registration phase, therefore it is out of our consideration. The throughput of blockchain based on PoW consensus mechanism has an upper limit, say the maximal throughput per second of blockchain in Yazdinejad’s scheme is maintained at about 7, which has greatly affected the efficiency of their scheme. In our scheme, the throughput of the blockchain equals to the number of requests, for that the throughput of RPBFT can be maintained at 1000TPS-100000TPS. Therefore, our solution can fully meet the demand for instantaneous throughput in practical application scenarios.

### Communication and storage overheads

In Tables 4 and 5, we present a comparison of the storage and communication overhead of the protocols. The output sizes for various cryptographic operations are as follows: SHA-256 hash (256 bits), RSA-1024 encryption/decryption (1024 bits), DSA-1024 signature (1024 bits), AES-128 symmetric encryption (128 bits), an ECC point (160 bits), and a random number (256 bits). Additionally, the lengths for the identity, password, and timestamp are all 32 bits.

**Table 4:** Comparison of storage overhead.

Scheme	Storage overhead		
	Server	Device (smart card)	Total
^8^	800bit	1312bit	2112bit
^18^	1536/1024bit	1536/1024bit	5120bit
^19^	288/288bit	544/544bit	1664bit
^22^	544/544bit	768/800bit	2656bit
Ours	0bit	1032/800bit	1832bit

**Table 5:** Comparison of communication overhead.

Scheme	Communication overhead verification
^8^	1920bit
^18^	7872bit
^19^	3200bit
^22^	1568bit
Ours	2784bit

Yazdinejad’s scheme^7^ does not provide specific storage and communication details, so its corresponding overhead cannot be accurately evaluated. In Xiang et al.’s scheme^8^, user devices interact with the medical server, which stores approximately 800 bits of user registration information on the blockchain. The smart card stores about 1312 bits of data. The communication overhead for registration and verification are approximately 1056 bits and 1920 bits, respectively. In Kumar et al.’s scheme^18^, patients and doctors use different devices, so in Table 4, we differentiate between the devices used by patients and doctors with a slash. During verification, the server forwards a large amount of encrypted data, resulting in a total overhead of approximately 7872 bits. In Chen et al.’s scheme^19^, we separately calculated the storage and communication overhead for user devices and sensors during registration. The total verification overhead is 3200 bits. In Mahmood et al.’s scheme^22^, we separately calculated the storage and communication overhead for user devices and sensors during registration. The total verification overhead is 1568 bits.

In the proposed scheme, the server (service provider) does not store additional user information for authentication during registration. The devices used by doctors and patients store 1032 bits and 800 bits of data, respectively. The communication overhead for registration is 1792 bits for doctors and 2368 bits for patients. The total communication overhead during the verification phase is approximately 5288 bits.

In terms of storage overhead, our scheme reduces the overhead by 2392 bits (a 13.3% reduction) compared to scheme^8^, and 3288 bits (a 64.2% reduction) compared to scheme^18^. It increases the overhead by 168 bits (a 10.1% increase) compared to scheme^19^, and 824 bits (a 31.0% reduction) compared to scheme^22^. The proposed scheme has 864 and 1216 more bits of communication overhead than schemes^8^ and^22^ respectively, and 5088 and 416 fewer bits than Schemes^18^ and^19^ respectively. To enhance communication security, our scheme additionally uses a key to encrypt the transmission content during the transmission process. Therefore, the communication overhead is slightly higher than that of schemes in^8^ and^22^.

**Fig. 7:**
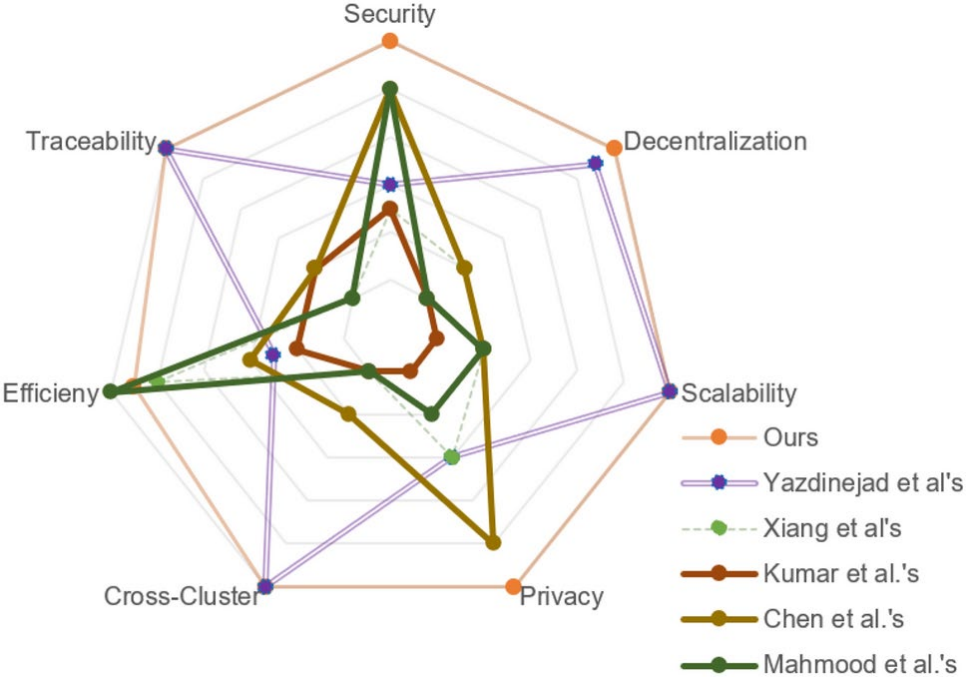
Comprehensive performance comparison.

Figure 7 comprehensively shows the advantages and disadvantages of six schemes. Therefore, our authentication protocol has higher security, efficiency, and privacy. Besides, it meets the needs of decentralization, scalability, and cross-cluster of medical scenarios.

The proposed system supports secure communication between doctors and patients across different hospitals, enabling integrated healthcare. Patient devices connected to sensors provide continuous health monitoring, with immediate alerts if critical thresholds are exceeded. Smart contracts ensure that only authorized entities access sensitive patient data, maintaining privacy and confidentiality. Implementation steps include setting up hospital nodes with independent registration centers and smart contracts. Doctors and patients register with their respective hospital nodes, verified by the registration center. Doctors send authentication requests to smart contracts, which are verified and processed in real-time, enabling secure communication with patients.

The proposed scheme demonstrates several practical advantages: (1) Efficiency: Low computational overhead and high TPS ensure the system can handle high volumes of authentication requests efficiently. (2) Scalability: The system can scale to accommodate additional hospital nodes without performance loss. (3) Security: Robust cryptographic mechanisms and consensus protocols ensure secure and reliable operations.

The proposed authentication and key agreement scheme, leveraging the alliance blockchain and RPBFT consensus mechanism, demonstrates high feasibility and operability for real-world medical applications. Its computational efficiency, scalability, security, and real-time operability make it a practical solution for secure and efficient healthcare communication. The system meets the demands of decentralized, scalable, and cross-cluster communication, ensuring robust and reliable operations in integrated healthcare networks.

## Conclusion

In this paper, we proposed a novel blockchain-based cross-hospital authentication scheme for IoMT-based healthcare. We realize efficient automatic authentication based on smart contracts and blockchain instead of relying on TTP. A novel DAS is proposed, and pseudo-identity is used to realize the anonymity of doctors and patients respectively. Based on these two strategies, we realize the privacy protection of doctors and patients meanwhile keep the records traceable. Based on the alliance blockchain and RPBFT consensus mechanism, we have realized a secure and efficient dynamic expansion of hospital nodes. Doctors and patients in different hospital nodes can communicate across clusters. We present formal security proof in the random oracle model to prove the semantic security of the proposed scheme, as well as security analysis of many other desirable properties. In addition, we conduct a simulation test on the scheme and compare it with two related schemes. The results and security analysis show that our scheme can achieve identity authentication, node expansion, and cross-hospital communication with lower computation overhead while ensuring security and privacy. Compared to most related schemes, our approach achieves a reduction of approximately 23% to 87% in computational overhead and a reduction of about 13% to 64% in storage overhead. The limitation of our scheme is that it increases the transmission volume due to the need for interaction with smart contracts in the blockchain, resulting in a transmission volume that is only at a moderate level compared to existing solutions. Future research can focus on the design of smart contracts specifically designed for secure and efficient cross hospital communication protocols.

## Data Availability

Data is provided within the manuscript.
